# Acceptability of capillary point-of-care testing: a systematic review

**DOI:** 10.1136/bmjopen-2025-115234

**Published:** 2026-09-15

**Authors:** Danielle April Dunnett, Cecilia Casetta, Ebenezer Oloyede, Emilia Loane, James MacCabe, David Taylor

**Affiliations:** 1 King’s College London Institute of Psychiatry Psychology & Neuroscience, London, UK; 2 South London and Maudsley NHS Foundation Trust, London, UK

**Keywords:** PUBLIC HEALTH, Systematic Review, Patient Satisfaction

## Abstract

**Abstract:**

**Objective:**

To identify and synthesise evidence on the acceptability and perceived experience of finger-prick point-of-care testing (POCT) among patients and clinicians across healthcare settings.

**Design:**

Systematic review conducted in accordance with Preferred Reporting Items for Systematic Reviews and Meta-Analyses (PRISMA 2020) guidelines.

**Data sources:**

Medline, Embase, PsycInfo, CINAHL, Cochrane and Web of Science were searched from inception to January 2024 and re-run in July 2025, supplemented by citation tracking of relevant studies.

**Eligibility criteria:**

Studies reporting patient and clinicians’ experiences, perceptions, satisfaction or acceptability relating to finger-prick POCT for any health condition or blood parameter were eligible. Quantitative, qualitative and mixed-methods designs were included.

**Data extraction and synthesis:**

Data were extracted independently by two reviewers and synthesised using thematic analysis and narrative synthesis. Methodological quality was appraised using the Mixed-Methods Appraisal Tool.

**Results:**

21 studies met the inclusion criteria, encompassing 9128 participants (17 quantitative, 3 qualitative, 1 mixed methods). Across diverse clinical contexts, finger-prick POCT was reported as generally acceptable, less distressing and perceived as a convenient alternative to venous sampling in comparative studies. Thematic synthesis identified two major themes: (1) enhancing the patient–clinician relationship through improved engagement, communication and understanding of care and (2) clinical implications of finger-prick POCT on clinicians’ workflow, confidence and skill acquisition. Finger-prick POCT was perceived to promote personalised consultations, enable immediate discussion of results and streamline decision-making. Clinicians highlighted its potential to expand task sharing, improve efficiency and strengthen continuity of care, although concerns regarding training, reliability and quality assurance were identified.

**Conclusions:**

Finger-prick POCT is generally acceptable to patients and clinicians, improving comfort, convenience, engagement and perceived efficiency. Implementation should prioritise training, infrastructure and quality assurance frameworks to maximise clinical and experiential benefits.

**PROSPERO registration number:**

CRD42024512130.

**STRENGTHS AND LIMITATIONS OF THIS STUDY**

- Comprehensive and systematic search across multiple databases covering diverse clinical settings and populations.
- Inclusion of both patient and clinician perspectives using mixed qualitative and quantitative designs.
- Conducted using a preregistered PROSPERO protocol and Preferred Reporting Items for Systematic Reviews and Meta-Analyses (PRISMA) 2020 reporting guidance.
- Methodological quality appraised using the Mixed-Methods Appraisal Tool to ensure rigour.
- Limited to English-language studies and adult populations, which may affect generalisability.

## Introduction

 Diagnostic testing plays a crucial role in clinical decision-making, particularly in ruling out diseases and guiding the initiation, modification or cessation of treatment, ultimately improving patient outcomes.^1 2^ In most healthcare settings, this process relies on venepuncture-based laboratory testing, an invasive procedure^3 4^ that requires coordination with laboratory services and follow-up, which may delay clinical decision-making and treatment optimisation.

The growing adoption of point-of-care testing (POCT), defined as diagnostic testing performed at or near the site of care, represents a shift towards a more decentralised and responsive model of healthcare delivery.^5^ POCT has been associated with reductions in turnaround times and hospital length of stay^6 7^ and is increasingly used across primary and hospital-based settings for a range of haematological and biochemical measurements.^8–11^ Devices may assess individual biomarkers such as glycated haemoglobin (HbA1c),^12^ glucose, lipid profiles,^13^ or creatinine,^14^ or use multiplex cartridges to measure multiple parameters simultaneously,^9^ thereby supporting more timely diagnosis and treatment decisions, while potentially improving workflow efficiency and patient management.^15 16^ Importantly, capillary and venous sampling differ not only in the method of collection but also in physiological sample composition and in preanalytical and analytical factors, including sample collection, handling and analytical performance characteristics, which may influence test performance, interpretation, clinician confidence and user experience.^17^

While POCT offers clear operational and clinical advantages, successful integration into routine care depends not only on analytical performance, but also on its acceptability to both patients and healthcare professionals. Acceptability is a multidimensional construct encompassing perceived burden, tolerability, convenience, trust in results and the extent to which a technology can be integrated into clinical workflows.^18^ As such, acceptability represents an important but relatively underexplored determinant of successful implementation.

The importance of acceptability is particularly evident in contexts requiring frequent or long-term blood monitoring, including cancer,^19^ infectious diseases^20^ and diabetes.^21^ More recently, finger-prick POCT has been introduced into mental health settings, particularly for monitoring clozapine treatment, where mandatory haematological monitoring requires repeated venous assessment of white cell count (WCC), absolute neutrophil count (ANC) and plasma clozapine levels.^22–24^ In these contexts, finger-prick POCT may reduce the burden associated with repeated venous testing, improve service accessibility and facilitate more timely clinical decision-making. These factors may in turn support treatment initiation, adherence and continuation.^22 23 25^

Although individual studies report high levels of satisfaction and preference for finger-prick POCT compared with traditional venous testing, citing improved tolerability and reduced stress,^26 27^ the existing evidence base remains fragmented and methodologically heterogeneous. Acceptability is variably defined and measured, often as a secondary outcome, and reported across diverse populations, clinical settings and testing modalities. Quantitative findings, such as preference rates and satisfaction scores, and qualitative insights, including patient and clinician experiences, are rarely synthesised in an integrated manner. In addition, while finger-prick testing may offer advantages for individuals with needle phobia, poor venous access or limited cooperation,^26 28 29^ concerns remain regarding analyser accuracy,^30^ regulatory compliance,^31^ calibration and quality assurance requirements^32^ and potential user-dependent variability associated with operator training and sampling technique,^33 34^ all of which may influence perceived acceptability in practice.

Previous reviews have typically focused on specific professional groups,^32^ healthcare settings^35^ or clinical conditions^15 36^ and have not comprehensively examined acceptability as a multidimensional construct across diverse clinical settings. Consequently, there remains a lack of consolidated evidence to inform the implementation of finger-prick POCT in routine clinical practice. This review addresses this gap by synthesising evidence from comparative and non-comparative studies using thematic analysis within a narrative synthesis framework to identify factors influencing the acceptability of finger-prick POCT among patients and healthcare professionals across diverse clinical settings.

## Methods

We conducted a systematic review to identify and synthesise studies examining the acceptability of finger-prick POCT among patients and healthcare professionals. POCT was defined as diagnostic testing performed at or near the site of care that provides results during the same clinical encounter without the need for laboratory processing. Studies were eligible if they evaluated finger-prick blood sampling either in comparison with venous sampling or in isolation, reflecting its use as an alternative to standard venepuncture-based pathways in routine care. Where comparative outcomes were assessed, venous sampling was specified as the comparator to ensure consistency in interpretation across studies. Acceptability encompassed perceptions, experiences, and attitudes towards testing, including domains such as ease of use, comfort, convenience, confidence in results, perceived burden, workflow impact and preference for sampling method, consistent with established conceptual frameworks of acceptability.^18^

### Protocol and registration

The study was preregistered with the International Prospective Register of Systematic Reviews (PROSPERO; registration ID: CRD42024512130) and was conducted in accordance with Preferred Reporting Items for Systematic Reviews and Meta-Analyses (PRISMA) guidelines for systematic reviews. The PRISMA checklists are available in online supplemental dataset 1, S3. Additionally, all changes made to the PROSPERO protocol are provided in online supplemental dataset, S4.

### Study eligibility criteria

Interventional and observational studies using quantitative, qualitative or mixed-methods designs were eligible for inclusion. Studies involving adult participants (>18 years) in any clinical setting, including inpatient and outpatient environments, were included. Only studies published in English were included due to feasibility and resource constraints. This restriction may have excluded relevant non-English studies and is acknowledged as a limitation.

#### Inclusion criteria

- Assessed acceptability, satisfaction, experience or preference relating to finger-prick POCT.
- Evaluated finger-prick sampling either:
  - In comparison with venous blood sampling.
  - In isolation, where finger-prick POCT was used as an alternative to standard venous-based testing pathways.
- Reported direct patient and/or clinician feedback using quantitative and/or qualitative methods.

#### Exclusion criteria

- Evaluated non-finger-prick capillary samples (eg, earlobe, heel, forearm).
- Focused exclusively on self-testing in home or non-clinical settings.
- Assessed acceptability indirectly (eg, uptake rates without user-reported experience).
- Used venous rather than finger-prick sampling despite evaluating POCT devices.
- Reported only incidental acceptability data (eg, a single pain or satisfaction outcome) where acceptability was not a substantive focus of the study and insufficient detail was provided to inform the review objectives.

Reviews, meta-analyses, conference papers, lecture notes and editorials were excluded; however, reference lists of reviews and meta-analyses were screened for eligible studies.

### Sources and search strategy

We searched Medline, EMBASE, PsycINFO, CINAHL, Cochrane Library and Web of Science from inception to January 2024. The search was updated in July 2025 to identify additional eligible studies published since the initial search. The search strategy was developed in collaboration with a librarian using a combination of MeSH terms and keywords. Search strings were piloted and iteratively refined to maximise sensitivity. Search terms combined keywords related to point-of-care testing and finger-prick sampling (eg, “point-of-care test*”, 'finger* prick”, “capillary test*'). The full search strategy is available in online supplementary data 1, S1. Additional studies were identified through backward citation tracking of included studies to ensure comprehensive coverage.

### Selection process

After deduplication, titles and abstracts were independently screened by two reviewers (DAD and CC) using Rayyan (Rayyan Systems, Cambridge, Massachusetts, USA; https://www.rayyan.ai). Full texts of potentially eligible studies were subsequently reviewed independently by the same reviewers. Disagreements were resolved through discussion with senior coauthors (JM and DT).

### Data extraction

Data were extracted using a standardised Microsoft Excel form by one reviewer (DAD) and independently checked by a second reviewer (CC), with discrepancies resolved through discussion with senior authors (JM and DT). The following data were extracted:

- Study characteristics (author, year, country, setting).
- Study design and sample size.
- Participant characteristics (age, sex).
- Clinical context and point-of-care devices used.
- Comparator intervention (where applicable).
- Measures of acceptability and user experience.
- Outcome measures and analytical methods.

Quantitative acceptability data extracted included:

- Proportion-based outcomes (percentage preference or satisfaction).
- Scaled outcomes (Likert-scale, Visual Analogue Scale (VAS) scores).
- Composite or validated measures assessing domains such as burden, convenience and satisfaction.

Where reported, both patient and clinician-reported outcomes were extracted. Qualitative data, including themes, narratives and participant quotations, were also extracted.

### Data synthesis

A narrative synthesis approach was used, incorporating thematic analysis of qualitative findings alongside descriptive synthesis of quantitative data. Full texts of included studies were uploaded to NVivo V.14 (https://lumivero.com/products/nvivo/).

Qualitative data, including participant quotations, narratives, and author interpretations, were analysed using Braun and Clarke’s^37^ six-phase framework, including (1) familiarisation; (2) initial coding; (3) theme development; (4) review and refinement; (5) defining and naming themes and (6) integration with supporting data. Two reviewers (DAD and EO) independently conducted the analysis. Regular discussions were undertaken to refine themes and ensure consistency in interpretation. A thematic map was developed to illustrate findings.

Quantitative findings were highly heterogeneous in terms of study design, populations, outcome measures, and reporting formats, including proportions, Likert-scale responses, VAS scores, and validated instruments assessing burden, convenience and satisfaction. Due to this heterogeneity, formal meta-analysis was not considered appropriate. Instead, quantitative findings were synthesised descriptively by: (1) grouping outcomes into key domains of acceptability (preference, pain/discomfort, satisfaction, convenience and psychological impact); (2) summarising ranges and patterns across studies and (3) examining the direction and consistency of findings across studies. Quantitative findings were then integrated within the thematic framework to support and contextualise qualitative themes. A structured summary of quantitative findings is presented in online supplementary dataset 1, S8.

### Integration of findings

Findings from qualitative, quantitative, and mixed-methods studies were integrated through narrative synthesis to identify recurring patterns relating to the acceptability of finger-prick POCT across diverse clinical settings.

### Quality assessment

The methodological quality of included studies was assessed using the Mixed-Methods Appraisal Tool, 2018 version (MMAT).^38^ Two reviewers (DAD and CC) independently conducted the appraisal in Microsoft Excel, resolving discrepancies through discussion. The MMAT evaluates core methodological domains across qualitative, quantitative and mixed-method designs, including study design, sampling, response rates, confounders, outcome measures and analysis. Each study was assessed against five criteria, with responses classified as ‘Yes’, ‘No’ or ‘Can’t tell’. Studies were rated as high (5/5), medium (4/5) or low quality (<3/5). All studies were included in the synthesis regardless of quality to ensure comprehensive integration (online supplementary dataset 1, S5 and S5.1).

### Patient and public involvement

Patients and/or the public were not involved in the design, conduct, reporting or dissemination plans of this review.

## Results

### Search results

**Figure 1:**
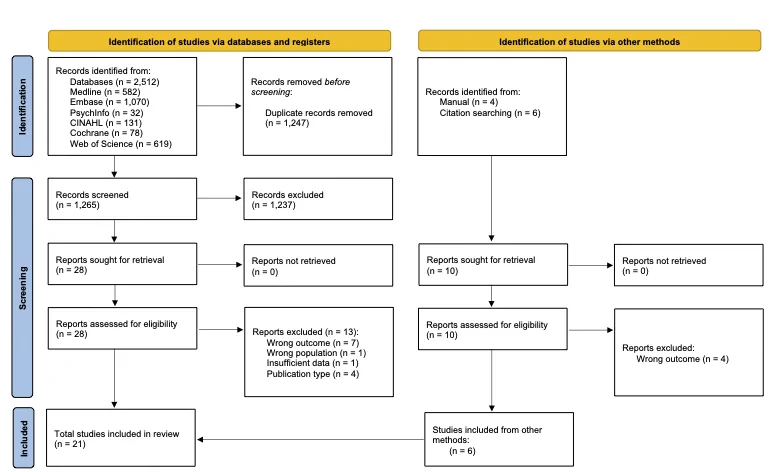
Preferred Reporting Items for Systematic Reviews and Meta-Analyses (PRISMA) flow diagram of search strategy and results.

A total of 2512 records were identified through database searches. After the removal of duplicates, 1265 records remained and were screened for eligibility based on their titles and abstracts. An additional 10 relevant studies^13 16 26–31 39 40^ were identified through citation searching of included studies and related reviews.^15 32^ Following full-text review of 38 articles, 21 met the eligibility criteria and were included in this systematic review^13 16 20–24 26–28 40–50^ (figure 1). Reasons for exclusion of the remaining 17 articles are detailed in online supplemental dataset 1, S2.

### Study characteristics

Among the 21 included studies, publication years ranged from 2003 to 2024. Sample sizes varied between 15 and 4968, with a total sample of 9128 participants. Among studies reporting participant sex, 5424 participants (59.4%) were male, and the mean age of participants ranged between 45 and 52 years. Of the included studies, 10 were conducted in outpatient settings,^20 22 26–28 42 43 45 48 49^ 5 included both inpatient and outpatient populations,^23 24 41 44 47^ 2 were conducted in early intervention service and community mental health team settings,^13 40^ 3 were conducted in general practice settings^16 21 50^ and 1 was conducted in a hospital-based setting.^46^ Three of the included studies were clinician-only populations.^40 46 50^

The blood parameters assessed in these studies included:

- Clozapine plasma levels (n=2).^22 24^
- International Normalised Ratio (INR) (n=4).^16 27 43 46^
- Glycaemic markers (HBA1c and glucose) (n=5).^13 16 21 40 42^
- Cholesterol (lipid panel) (n=3).^13 16 40^
- Creatinine (n=1).^42^
- HIV (CD4 count) (n=5).^44–49^
- Hepatitis (n=1).^20^
- WCC and ANC (n=3).^23 26 41^
- Haematocrit (HCT) and haemoglobin (HGB) (n=1).^28 48^

As multiple parameters were measured in several studies, some studies were counted more than once.^13 16 40 42 48^ Geographically, the majority of studies were conducted in the UK (n=6)^13 22 40 42 45 50^ or Australia (n=4).^16 20 46 49^ In terms of study design, there were 17 quantitative studies,^16 20–24 26–28 41–45 47–49^ 3 qualitative studies^40 46 50^ and 1 mixed-methods study.^13^ Additionally, two studies explored hypothetical access to capillary POCT for INR and viral and bacterial infections.^46 50^ Table 1 provides a summary of the study characteristics of included studies. Detailed study characteristics, including participant demographics, are provided in online supplementary dataset 1, S6.

**Table 1:** Summary characteristics of included studies

Study	Year	Country	Setting	Clinical parameter	POCT device	Sample size	MMAT
Bajis *et al*^20^	2018	Australia	Outpatient	Hepatitis C virus	Xpert HCV	565	●●●◯◯
Bogers *et al*^41^	2015	Netherlands	Mixed inpatient/outpatient	WCC, ANC	HaemoCue	93	●●●◯◯
Kelly *et al*^23^	2021	Australia	Mixed inpatient/outpatient	WCC, ANC	AthelasOne	40	●●●●●
Kamhi-Nesher *et al*^24^	2022	Israel	Mixed inpatient/outpatient	Clozapine levels	MyCare Insite	50	●●●●●
Nielsen *et al*^26^	2012	Denmark	Outpatient	WCC, granulocytes	Chempaq XBC	151	●●●◯◯
Atkins *et al*^22^	2022	UK	Outpatient	Clozapine levels	MyCare Insite	116	●●●●●
Woods *et al*^27^	2004	Canada	Outpatient	INR	CoaguChek-S	60	●●●●●
Butler *et al*^13^‡	2020	UK	EIS/CMHT	Lipid, HBA1c	Afinion 2	29	●●●●●
Butler et al*^40^†	2021	UK	EIS/CMHT	Lipid, HBA1c	Afinion 2	15	●●●●●
Riva *et al*^43^	2020	Malta	Outpatient	INR	CoaguChek-XS	313	●●●●●
Yonel *et al*^42^	2022	UK	Outpatient	HBA1c, creatinine, Vit D	Nova StatSensor	158	●●●●●
Smits *et al*^21^	2022	Netherlands	General practitioners	HbA1c, Glucose	Afinion 2	169	●●●●◯
Laurence *et al*^16^	2010	Australia	General practitioners	Lipid, HbA1c, INR	DCA 2000	4968	●●●●◯
Daneau *et al*^48^	2016	Belgium	Outpatient	CD4, ALT, HGB	Pima CD4	499	●●●◯◯
Conway *et al*^49^	2015	Australia	Outpatient	CD4, viral load HIV	Determine Combo Assay	1093	●●●●◯
Glover et al*^46^†	2008	Australia	Hospital-based	INR	Hypothetical POCT	33	●●●●●
Butler et al*^50^†	2008	UK	General practitioners	Viral, bacterial	Hypothetical POCT	40	●●●●●
Gauthier *et al*^47^	2012	France	Mixed inpatient/outpatient	CD4, viral load	RHT VIKIA	455	●●●●◯
Herbert *et al*^45^	2012	UK	Outpatient	CD4	PIMA CD4	283	●●●●◯
Liu *et al*^44^	2003	Thailand	Mixed inpatient/outpatient	HIV	Haema Strip	804	●●●●◯
Pourafshar *et al*^28^	2024	USA	Outpatient	HCT, HGB	BD MiniDraw	113	●●●●●

Detailed study and participant characteristics are provided in online supplementary dataset 1, S6.

* Clinician-only studies.

† Qualitative.

‡ Mixed-methods.

ALT, alanine aminotransferase; ANC, absolute neutrophil counts; CD4, clusters of differentiation 4; CMHT, community mental health team; EIS, early interventions service; HbA1c, glycated haemoglobin; HCT, haematocrit; HGB, haemoglobin; INR, international normalised ratio; MMAT, Mixed-Methods Appraisal Tool; POCT, point-of-care testing; WCC, white cell count.

### Quality assessment of studies

All included studies (21/21, 100%) received a consolidated quality score of 3 or higher. For the mixed-methods study,^13^ separate quality assessments were conducted for its qualitative and quantitative components, as required by the MMAT. A detailed assessment of each study’s methodological quality using the MMAT is provided in online supplementary dataset 1, S5 and S5.1.

### Quantitative findings

Quantitative findings were reported using a range of measures, including proportions, Likert-scale responses, VAS scores, and validated satisfaction and convenience instruments. Due to heterogeneity in outcome measures and reporting formats, findings were synthesised descriptively rather than pooled statistically.

Across studies reporting preference, most participants generally favoured finger-prick sampling over venepuncture, with preference rates ranging from approximately 54% to 88%.^22 24 26 45^ Studies using VAS and Likert measures generally reported lower pain, reduced perceived burden and greater convenience associated with finger-prick sampling compared with venous testing.^16 23 26 27 41–43^ Overall, patient satisfaction with finger-prick POCT was high across studies, with reported satisfaction ranging from 96.0% to 100%.^21 44 49^ However, some studies identified concerns relating to device reliability, repeated sampling, device maintenance and workflow burden during early implementation.^13 21 40 48^ A structured summary of quantitative findings is presented in online supplementary dataset 1, S8.

### Themes

Two overarching themes were identified relating to the acceptability of finger-prick POCT and its implications for clinical implementation. Supporting participant quotations are presented in table 2, with full data available in online supplementary dataset, S7. The first theme, enhancing the patient–clinician relationship through finger-prick POCT, encompassed patient and clinician perceptions of engagement, communication and the care experience. The second theme, clinical implications of finger-prick POCT for clinician workflow, confidence and skill acquisition, explored implementation-related considerations. Themes and subthemes are summarised below, with interrelationships illustrated in the thematic map (table 3).

**Table 2:** Themes, subthemes and illustrative participant quotes

Themes	Subthemes	Source	Supporting quotes
Theme 1: Enhancing the patient–clinician relationship through finger-prick POCT: perceptions of engagement, communication and care experience	Subtheme 1.1: Improved care, communication and understanding of health management and treatments	Atkins *et al*, 2022 (P)^22^ Butler *et al*, 2021 (C)^40^ Butler *et al,* 2008 (C)^50^	[P] ‘Better to know results straightaway’ ‘advice on the spot’ ‘Helped with my understanding’ ‘Useful to know, as when I receive a text with my results, it’s just numbers and I can’t ask anything’ [C] ‘She had been in the team for a long time, and she would never want to get her bloods done [via venous], she always refused and then she said OK I will have it done, and so for her to actually change her attitude was brilliant’ [C] ‘We could explain to the patients…antibiotics are not going to work, which is justified by the results, as patients want the evidence as well’
	Subtheme 1.2: Perceived experience, preferences and tolerability of finger-prick POCT	Nielsen *et al,* 2012 (P)^26^ Atkins *et al*, 2022 (C)^22^	[P] ‘The capillary test is less painful’ ‘I prefer regular blood sampling because that is what I am used to’ [C] ‘Much easier to obtain a sample from patients with obscure veins’ ‘Able to take immediate action or escalate if required’
	Subtheme 1.3: Psychological impacts and perceived convenience of finger-prick testing	Bogers *et al*, 2015 (P)^41^ Glover *et al*, 2008 (C)^46^	[P] ‘Causes more anxiety [venous testing]’ ‘Is convenient, can be done anywhere, by your own nurse’ ‘When I was psychotic capillary sampling would have helped me a lot’ [C] ‘It’d be great for patient advocacy and empowering them to take some responsibility for their own health care’
Theme 2: Clinical implications of finger-prick POCT for clinician workflow, confidence and skill acquisition	Subtheme 2.1: Finger-prick POCT errors, accuracy and maintenance	Butler *et al*, 2020 (C)^13^ Butler *et al*, 2021 (C)^40^	[C] ‘Too many errors left the patient waiting; it seemed unprofessional’ [C] ‘Accuracy is probably not as fabulous as sending to the lab’ ‘I didn’t know how to correct what I’d done wrong, so I didn’t want to put them through the same situation again’
	Subtheme 2.2: POCT training and skill requirements, workload constraints and sample turnaround time	Butler *et al*, 2008 (C)^50^ Butler *et al*, 2021 (C)^40^	[C] ‘I can imagine there will be a lot of implications in maintaining this equipment with calibration and all the things that go with maintaining near-patient testing’ [C] ‘The more you do it, the more confident you are in reassuring patients and getting results’ ‘Because of the amount of time that I will spend chasing results, I’ll just go and do it myself [on the POC device] and get the results there and then. So, it’s making my work a lot easier’.

Participant types: P=patients; C=clinicians. Detailed participant quotations are provided in online supplementary dataset 1, S7.

POCT, point-of-care testing.

**Table 3:** Thematic map of included studies

Study (author; year)	T1. Enhancing the patient–clinician relationship through finger-prick POCT: perceptions of engagement, communication and care experience			T2. Clinical implications of finger-prick POCT for clinician workflow, confidence and skill acquisition	
	1.1. Improved care, communication and understanding of health management and treatments	1.2. Perceived experience, preferences and tolerability	1.3. Psychological impacts and perceived convenience of finger-prick testing	2.1. Finger-prick POCT errors, accuracy and maintenance	2.2. POCT training and skill requirements, workload constraints and turnaround time
Bajis *et al*., (2018)^20^		*			
Bogers *et al*., (2015)^41^		*	*		
Kelly *et al*., (2021)^23^	*	*	*	*	
Nesher *et al*., (2022)^24^		*			
Nielsen *et al*., (2012)^26^		*	*		
Atkins *et al*., (2022)^22^	*	*			*
Woods *et al*., (2004)^27^		*			
Butler *et al*., (2020)^13^	*	*	*	*	
Butler *et al*., (2021)^40^	*	*		*	*
Riva *et al*., (2020)^43^		*	*	*	
Yonel *et al*., (2022)^42^		*		*	
Smits *et al*., (2022)^21^	*	*		*	*
Laurence *et al*., (2010)^16^	*	*	*		
Daneau *et al*., (2016)^48^		*			
Conway *et al*., (2015)^49^		*	*		
Glover *et al*., (2008)^46^		*	*	*	*
Butler *et al*., (2008)^50^	*	*		*	*
Gauthier *et al*., (2012)^47^		*			
Herbert et al., (2011)^45^		*			*
Liu *et al*., (2003)^44^		*			
Pourafshar *et al*., (2024)^28^		*			

*Indicates that the study contributed data to the corresponding theme/subtheme.

POCT, point-of-care testing.

#### Theme 1: enhancing the patient–clinician relationship through finger-prick POCT

Included studies explored aspects of patient and clinician experiences of finger-prick POCT, including perceived pain, convenience, immediacy of results, patient management and satisfaction.^13 16 20–24 26–28 40–50^ This theme comprised three subthemes:

1. Improved care, communication, and understanding of health management and treatments.
2. Perceived experience, preferences and tolerability.
3. Psychological impacts and perceived convenience of finger-prick testing.

### Improved care, communication and understanding of health management and treatments

Seven studies^13 16 21–23 40 50^ reported that finger-prick POCT was perceived to improve aspects of clinical care by supporting patient–clinician interactions and enabling real-time discussion of results during consultations. The immediate availability of results was reported to provide a unique opportunity for clinicians to discuss findings in real time, strengthened patients’ understanding of their condition, facilitate shared decision-making and support more timely treatment adjustments.^13 16 21 22^

Delays inherent in venous testing workflows were reported to undermine patient confidence, prolong uncertainty and limit opportunities for timely clarification.^13 22^ Patients frequently reported difficulty interpreting results communicated via text message or outside of the clinical context, leaving them disengaged from treatment decisions. By eliminating these delays, finger-prick POCT was associated with quicker treatment adjustments, enhanced quality of communication during appointments, and fewer follow-up visits, outcomes valued by both patients and clinicians.^16 21 22 40^ Importantly, finger-prick POCT was highlighted as a critical facilitator for patients with needle phobia or challenging venous access, offering a less invasive, more tolerable procedure that improved both uptake and adherence to monitoring requirements.^21–23 40^ Clinicians reported leveraging finger-prick POCT results as powerful tools for clinical explanation, particularly in infectious disease management, where immediate, tangible evidence strengthened the rationale for treatment decisions and improved patient comprehension.^40 50^ The real-time availability of results was described as accelerating prescribing decisions, enabling medication adjustments within the same consultation and reducing dependence on external laboratories and delayed result processing.^22^

### Perceived experience, preferences and tolerability

Across multiple health conditions and clinical settings, studies reported patients generally preferred finger-prick POCT over traditional venepuncture, citing higher satisfaction, lower pain and greater procedural acceptability.^13 16 20–24 26–28 40–50^ Participants commonly described finger-prick testing as less invasive, quicker, and more convenient, with many explicitly describing it as a more patient-centred approach to blood monitoring. Six studies specifically assessed finger-prick POCT for bloodborne infections, including HIV and hepatitis.^20 44 45 47–49^ The immediacy of results was highly valued; 80% of patients indicated that receiving results during the same consultation improved their care experience and nearly all (96.8%) rated the procedure as satisfactory and less painful than venepuncture.^47 49^ Finger-prick POCT was often preferred over laboratory-based testing pathways, reinforcing its potential to increase engagement in testing and adherence to monitoring requirements.^20 44 45^ While one study noted that approximately 30% of patients reported postprocedural pain after three consecutive finger-pricks, prompting some to reconsider their preference.^48^

Clinician perspectives broadly aligned with these findings, although some expressed concern about blood exposure and contamination risk in infectious disease settings,^48^ most reported that finger-prick POCT improved workflow efficiency and simplified result delivery. For example, 61.2% of clinicians reported being satisfied with finger-prick POCT in HIV care, and 77.6% reported that results reporting was easier compared with venous sampling.^47^

Three studies explored demographic predictors of preference for finger-prick testing,^20 44 45^ revealing that female sex,^20^ younger age groups (26–35 years),^44^ higher educational attainment, and household income were all associated with greater preference for finger-prick testing.^44^ Patients with higher CD4 counts were also more likely to prefer finger-prick POCT, and preferences varied by ethnicity, with 74% of Black African participants preferring finger-prick testing compared with 50% of white participants and 32% of those from other ethnic groups.^45^

Seven studies evaluated finger-prick POCT in mental health settings, including mandatory full blood count monitoring for clozapine,^23 26 41^ therapeutic drug monitoring^22 24^ and routine physical health screening (lipid panel and HbA1c).^13 40^ Clinicians reported that finger-prick POCT improved patient engagement, particularly among patients who were non-cooperative with venous sampling, reducing the time required to obtain samples^23 41^ (12 min for finger-prick sampling compared with 21 min for venepuncture).^41^ Clinicians valued the ability to avoid laboratory coordination, make immediate treatment adjustments and reduce delays in care. Importantly, studies suggested that availability of finger-prick testing may support clozapine initiation and adherence, underlining its potential to increase access to this critical therapy.^23^

Patients receiving clozapine treatment described finger-prick POCT as beneficial, emphasising the perceived convenience of in-clinic sampling by their own clinician and the reassurance provided by immediate results, which together helped reduce anxiety associated with off-site phlebotomy.^23^ While a minority preferred venepuncture due to familiarity,^26^ overall satisfaction with finger-prick POCT was high. Two studies assessed finger-prick POCT for clozapine therapeutic drug monitoring using the MyCare Insite analyser in 151 patients.^22 24^ Among clinicians, 81% preferred finger-prick POCT over standard venous approaches,^24^ and the majority of patients rated their experience as ‘good’ or ‘very good’ (77% and 88%, respectively).^22^

Clinicians noted the practical advantages of obtaining samples from patients with small or obscure veins and emphasised the usefulness of immediate results in guiding clinical decisions.^24^ In another study involving psychiatrists, nurses, and allied health professionals, finger-prick POCT facilitated early identification of metabolic abnormalities, supported collaborative care planning with patients and enabled non-phlebotomy staff to take a more active role in routine physical health monitoring.^40^

Five studies examined finger-prick POCT for INR testing in conditions including diabetes, chronic atrial fibrillation and anticoagulant management.^16 21 27 43 46^ These studies employed questionnaires,^16 21 27 43^ a VAS^27^ and semistructured group discussions,^46^ assessing various analysers (CoaguChek-S, CoaguChek XS Plus, Affinion 2 and AccuChek Inform II)^16 21 27 43^ and hypothetical finger-prick POCT for warfarin management.^46^ Across studies, patients expressed greater satisfaction and a preference for finger-prick POCT over venepuncture,^16 27 43^ citing reduced pain^27^ and shorter procedural times (42 min for finger-prick vs 75 min for venous sampling; p<0.001).^21 27^ Notably, patient satisfaction was positively correlated with age, suggesting that older adults may derive particular benefit from finger-prick approaches.^43^

Clinicians and pharmacists echoed these findings, describing traditional laboratory pathways as inefficient (‘very clumsy with lots of faults’) and endorsing finger-prick POCT as a strategy to improve accessibility and turnaround times for results.^46^ General practitioners further emphasised the importance of integrating finger-prick POCT systems with electronic health records to enable seamless care coordination.^46^ Three additional studies extended these findings to non-communicable diseases,^42^ common infections^50^ and general population screening.^28^ Patients rated venepuncture as more painful both during and after the procedure compared with finger-prick testing^42^ and clinicians valued finger-prick POCT for infectious disease management for its ability to provide immediate evidence and support treatment decisions.^50^ In a general population study, 65% of participants preferred finger-prick sampling, and 73% reported high satisfaction with the time taken to collect the sample.^28^

### Psychological impacts and perceived convenience of finger-prick testing

Across multiple studies, patients described finger-prick blood testing as less psychologically burdensome than venepuncture, with lower reported levels of stress, anxiety, worry and procedural discomfort.^13 16 23 26 41 43 46 49^ In a study using the Athelas One POCT device for clozapine monitoring, patients described greater worry and concern about venous blood draws, reinforcing the potential of finger-prick POCT to alleviate monitoring-related anxiety.^23^ Similarly, Bogers *et al* found significantly heightened pretest worry and fear associated with venous blood collection, although a minority of patients (7%) reported initial anxiety with finger-prick sampling, largely attributable to familiarity with venous methods.^41^

Convenience was consistently identified as a major factor influencing acceptability. Nielsen *et al*^26^ reported 87.1% of treating teams considered finger-prick POCT a more convenient option for patients,^26^ and other studies demonstrated lower scores for psychological burden and perceived ‘hassle’ when finger-prick testing was used.^16 43^ Patients also reported finger-prick POCT improved motivation to attend monitoring appointments, while clinicians highlighted its role in promoting patient empowerment and active engagement with monitoring requirements.^46^

Importantly, the perceived burden of venous sampling was greater for inpatients compared with outpatients, yet both groups reported a preference for finger-prick sampling.^41^ Some patients reported mild anxiety with their first finger-prick test, but this did not exceed typical pretest anxiety associated with venous collection and tended to diminish with familiarity.^13^ Overall, many patients described feeling less stressed and more reassured when finger-prick testing was available,^49^ appreciating both its less invasive nature and the ability to avoid laboratory visits and interactions with unfamiliar staff.^13 26^

#### Theme 2: clinical implications of finger-prick POCT for clinician workflow, confidence and skill acquisition

Six studies explored the influence of finger-prick POCT on clinical workflow, clinician capacity and confidence, implementation, decision-making and skills development.^13 21 22 40 49 50^ Two subthemes emerged:

1. Finger-prick POCT errors, accuracy and maintenance.
2. POCT training, skill requirements, workload constraints and turnaround time.

### Finger-prick POCT errors, accuracy and maintenance

Several studies reported clinicians’ concerns regarding the use and implementation of finger-prick POCT, particularly in relation to device procedural errors, calibration requirements and device maintenance.^13 21 40 50^

Clinicians described their experiences with errors when using finger-prick POCT, noting that these disruptions in workflow affected patient coordination, therapeutic relationships and clinician confidence. Some clinicians felt they appeared unprofessional or incompetent when errors occurred, particularly in the initial stages of use. These concerns were exacerbated by their inability to explain or rectify errors, which led some to disengage from finger-prick POCT or abandon its use altogether.^13 40^ Additionally, clinicians noted that extended consultations caused by repeated attempts to obtain a valid result were frustrating and unfair to patients.

Patients age and experience also appeared to influence perceptions of finger-prick POCT device reliability. Younger patients, particularly men aged 18–29, were significantly more likely to express concerns about test accuracy than older individuals (27.1% vs 16.9%, p<0.01).^42 43^ Despite this, concerns about reliability were expressed by only a minority of patients overall (21.5%), with more (56.9%) reporting confidence in the accuracy of finger-prick POCT results. Pharmacists raised parallel concerns regarding the comparability of finger-prick POCT to laboratory-based methods, underscoring the importance of robust external quality control procedures to maintain reliability and safeguard patient safety.^46^

### POCT training, skill requirements, workload constraints and turnaround time

Studies reported that clinician training, experience, and familiarity with finger-prick POCT influenced both perceived competence and confidence in using the devices effectively.^21 40 45 50^ A quantitative survey found that 97% of nurses felt they had sufficient skills to perform HbA1c and 100% for glucose testing. Reported confidence in test results was similarly high, 88% for HbA1c and 100% for glucose.^21^ Clinicians with regular POCT use described it as a means of acquiring new skills and enhancing professional development.^40^ Some clinicians viewed finger-prick testing as a component of holistic patient care and linked it to a broader clinical skillset.^40^ However, others reported limited opportunities to practice these skills following initial training, which affected confidence and consistency in performing finger-prick POCT.^40^

Time constraints and workload demands were commonly cited as barriers to implementation and routine use of finger-prick POCT. Clinicians described the additional time required for device preparation, calibration, quality assurance and result processing as contributing to prolonged consultation times.^21 50^ In some cases, these demands were perceived as burdensome. However, once integrated into routine clinical workflows, some clinicians perceived finger-prick POCT as improving efficiency by enabling immediate clinical decision-making and reducing the need to follow up on laboratory results.^40^ Clinicians generally agreed that finger-prick testing devices could be operated by any healthcare professional with appropriate training and cognitive ability.^46^ However, some clinicians considered nurse prescribers were best positioned to perform finger-prick POCT due to their ability to act on test results and initiate treatment without delay.^46^

Barriers to clinician confidence were attributed to limited access to evidence supporting finger-prick POCT accuracy, inadequate practical experience and insufficient mentorship. Clinicians with greater exposure to real-world troubleshooting scenarios reported feeling more capable of addressing patient concerns and interpreting results accurately.^40 46^ Despite these challenges, most clinicians recognised the broader value of finger-prick POCT. A majority expressed an interest in continuing POCT use, with 83% of nurses supporting its use for HbA1c testing, 59% for glucose testing and more than half reporting that finger-prick POCT was perceived to contribute to increased job satisfaction (HbA1c 62%, glucose 59%).^21^

## Discussion

These findings suggest that, when supported by appropriate training, infrastructure, and quality assurance processes, finger-prick POCT is generally acceptable and often preferred over venous sampling in comparative studies across diverse clinical settings. Across the 21 included studies, finger-prick testing was associated with greater patient satisfaction, lower perceived procedural burden and improved engagement with monitoring and care processes. Clinicians frequently described finger-prick POCT as supporting more timely clinical decision-making, streamlining aspects of workflow and facilitating testing in patients who may otherwise struggle to engage with conventional venepuncture pathways. Collectively, these findings suggest that finger-prick POCT may represent an acceptable alternative to venous sampling in many routine monitoring contexts, with potential to improve aspects of routine monitoring and care delivery.

From the patient perspective, finger-prick testing was generally viewed more favourably than venepuncture, with many studies reporting preferences for finger-prick testing, although this was not consistent across all populations and settings. Participants commonly described finger-prick sampling as less invasive, convenient and associated with lower levels of anxiety and distress.^26 27 44 45 47 49^ These characteristics appeared to contribute to a more tolerable testing experience and may support engagement with ongoing monitoring requirements. Several studies also reported that the immediate availability of results promoted patient involvement in consultations and facilitated discussion regarding treatment and disease management.^16 46^ Real-time feedback improved patients’ comprehension of treatment.^13^

The potential value of finger-prick POCT appeared particularly relevant in mental health settings. For some patients receiving clozapine treatment, particularly those experiencing paranoia, psychosis, needle phobia or difficulties tolerating venous sampling, conventional phlebotomy was described as distressing and invasive, creating barriers to frequent monitoring and to clozapine treatment.^23 31^ In these contexts, finger-prick testing was perceived as a more acceptable and practical alternative that could reduce resistance, support monitoring adherence and facilitate timely treatment adjustments.^23 41^ These findings suggest that finger-prick POCT may help reduce barriers to monitoring in psychiatric services and may support access to treatments requiring regular blood testing, such as clozapine.

Several patient-level factors appeared to influence preferences for finger-prick POCT. Some studies reported greater preference among women, older adults, individuals with higher educational attainment and income and patients with chronic illness.^43–45^ One study also reported greater preference for finger-prick testing among Black patients compared with white or other ethnic groups.^45^ However, these findings were not consistent across all studies and should be interpreted cautiously given differences in study populations and methodologies.

Despite generally favourable perceptions, not all patients preferred finger-prick testing. Some patients, particularly those accustomed to routine venepuncture, reported finger-prick testing as unfamiliar and anxiety-inducing.^26^ For these individuals, established routines and familiarity outweighed finger-prick POCT benefits.^41^ However, even among this group, there was recognition that finger-prick testing could have been valuable during periods of acute illness.^41^ One study also reported that repeated finger-prick procedures could result in post-procedural discomfort that influenced subsequent preference.^45^ These findings suggest that patient preferences are likely influenced by previous healthcare experiences, clinical context and individual expectations regarding testing procedures.

Clinicians’ perspectives on finger-prick POCT were more variable. While many clinicians recognised its potential to facilitate timely decision-making, reduce dependence on laboratory services and improve efficiency, concerns relating to device reliability, calibration, maintenance requirements and workflow disruption, particularly during initial implementation, were also frequently reported.^13 21 40 46 50^ Less experienced users were more likely to report these issues, especially when early device errors undermined confidence or rapport. Additionally, difficulties integrating POCT results into existing clinical information systems were seen as a barrier to coordinated care.^46^ Collectively, these findings suggest that acceptability among clinicians is influenced not only by patient experience but also by confidence in device reliability, governance and integration within existing clinical workflows.

Additional concerns were identified in infectious disease settings, where some clinicians expressed reservations regarding blood exposure and contamination risk during finger-prick sampling.^48^ Other implementation challenges included the need for frequent calibration, quality assurance procedures and ongoing staff training.^32 50^ Although the issues were not the primary focus of most included studies, they represent important contextual considerations for implementation and may contribute to variability in clinicians’ perceptions of finger-prick POCT.

Despite these challenges, many clinicians considered implementation barriers to be manageable with appropriate training, technical support, governance structures and clear diagnostic evidence. Several studies reported that clinicians valued finger-prick POCT clinical benefits,^13 46 49 50^ including improved access to testing, supporting patient engagement, enabling more rapid treatment decisions and facilitating monitoring among hard-to-engage populations, who would otherwise refuse monitoring through conventional pathways.^21–23 25 41^ Clinicians also described potential benefits relating to workflow efficiency, professional development and patient-centred care.^20 37 43^

Most included studies were conducted in outpatient settings, with comparatively fewer studies evaluating finger-prick POCT in inpatient environments. Although inpatient settings may present opportunities where POCT is particularly valuable, the relative paucity of inpatient acceptability studies limits understanding of how user experience and implementation may differ across healthcare settings. Further research comparing acceptability and implementation in inpatient and outpatient contexts would therefore be valuable.

Although finger-prick was generally viewed positively, several broader operational considerations should be acknowledged, POCT is frequently performed by non-laboratory staff, which may increase susceptibility to user-dependent variability, particularly during preanalytical stages such as sample collection and handling. Variability in operator training, adherence to standardised procedures and familiarity with device troubleshooting may also contribute to inconsistent performance and influence clinician confidence in POCT implementation. Furthermore, POCT operates within a different quality assurance framework from centralised laboratory testing, with implications for calibration, governance, standardisation and external quality control procedures. While these issues were not systematically evaluated within the included studies, they represent important considerations when interpreting acceptability findings and planning wider implementation.

### Methodological and systematic review strengths and limitations

This review’s strength lies in its comprehensive synthesis of qualitative and quantitative evidence on the acceptability of finger-prick POCT across diverse clinical contexts, populations and testing applications. By integrating findings from diverse study designs, the review was able to identify common themes relating to patient experience, clinician perceptions, convenience, procedural burden and implementation challenges that are critical to real-world implementation and sustainability. The inclusion of mental health settings, often underrepresented in diagnostic implementation research, adds unique value, offering insight into populations where venous sampling may represent a significant barrier to engagement with care. The review additionally focused specifically on acceptability from both patient and clinician perspectives, an important but comparatively unexplored aspect of POCT implementation. Identifying both facilitators and barriers to implementation may help inform future service development, staff training, workflow integration and implementation strategies across healthcare settings.

However, several limitations should be acknowledged. First, the review included only English-language studies involving adults. This restriction may have introduced language bias and limited generalisability to non-English-speaking settings or paediatric populations. Second, although the review was not restricted to comparative studies and studies evaluating finger-prick POCT in isolation were eligible for inclusion, much of the available literature assessed finger-prick POCT relative to venous sampling. Consequently, the overall synthesis was predominantly informed by comparative perceptions of capillary versus venous testing, which may differ from studies evaluating finger-prick POCT as a standalone testing pathway. Third, methodological quality varied across studies, and several studies were limited by small sample sizes, single-centre designs or heterogeneous outcome measures. Although all studies meeting the eligibility criteria were included to maximise breadth of evidence, inclusion of lower-quality studies may affect the strength of conclusions. Fourth, studies in which acceptability was reported only incidentally, for example, as a single pain or satisfaction outcome within studies primarily evaluating diagnostic performance or device reliability, were excluded. Consequently, some narrower aspects of acceptability may not have been captured, which may limit the breadth of the available evidence. Finally, a proportion of included studies were identified through citation tracking and supplementary searching, suggesting that some relevant studies may not have been fully captured through database searching alone.

Additionally, although many studies reported quantitative measures of acceptability, substantial heterogeneity existed in measurement tools, study populations, outcome definitions and reporting formats. Formal meta-analysis was therefore not considered appropriate, as pooled estimates may have been misleading or difficult to interpret meaningfully across such diverse study designs and clinical contexts.

### Implications and conclusion

Finger-prick POCT was generally acceptable across a range of healthcare settings and was often preferred over traditional venous sampling in comparative studies. Patients commonly reported greater convenience, lower procedural burden and reduced anxiety, while clinicians highlighted potential benefits relating to workflow efficiency, real-time clinical decision-making and patient engagement. However, acceptability was not universal, and implementation challenges relating to reliability, quality assurance, workflow integration and staff training remain important considerations. Several studies suggested that finger-prick POCT may support engagement with monitoring and treatment pathways, particularly among populations who experience difficulties with conventional venepuncture. Even modest improvements in treatment engagement or monitoring adherence may have implications in settings involving long-term or high-burden monitoring requirements. For these individuals, even small gains in adherence can yield meaningful outcomes. Given the increasing use of decentralised diagnostic technologies, further evaluation of the long-term implementation, acceptability and sustainability of finger-prick POCT across diverse healthcare settings is warranted. Future research should examine implementation in broader populations and settings, while also evaluating diagnostic performance, workflow integration, cost-effectiveness and long-term clinical outcomes. Further work may be particularly valuable in mental health settings, where finger-prick POCT has potential to reduce barriers to monitoring and support continuity of care for patients requiring regular blood testing.

## Supplementary material

10.1136/bmjopen-2025-115234 online supplemental file 1

## Data Availability

All data relevant to the study are included in the article or uploaded as supplementary information.
