# Supplementary material for: Acceptability of capillary point-of-care testing: a systematic review
Source: BMJ Open. 2026 Sep 15;16(9):e115234. doi: 10.1136/bmjopen-2025-115234 (PMC13583689; doi:10.1136/bmjopen-2025-115234)
Supplement: Supplementary file 1 [file bmjopen-16-9-s001.pdf]

Acceptability of Capillary Point-of-Care Testing: A Systematic Review.

Supplementary Dataset 1

Danielle A. Dunnett[1,]

Dr Cecillia Casetta[1]

Dr Ebenezer Oloyede[2]

Emilia Loane[1]

Prof James MacCabe[1]

Prof David Taylor[2]

[1] King's College London, Institute of Psychiatry, Psychology and Neuroscience, London, UK

[2] South London and Maudsley NHS Foundation Trust, London UK

To whom correspondence should be addressed:

Danielle A. Dunnett, King's College London, Institute of Psychiatry, Psychology and Neuroscience London, UK; e-mail: Danielle.2.Dunnett@kcl.ac.uk

Contents

Page 1 – 5: Supplementary 1 (S1): Detailed database search strings.

Page 6 – 8: Supplementary 2 (S2): List of excluded studies at full-text screening

Page 9 - 13: Supplementary 3 (S3): PRISMA 2020 checklists

Page 14: Supplementary 4 (S4): Changes from original PROSPERO protocol

Page 15 – 18: Supplementary 5 (S5) and 5.1 (S5.1): Quality assessment of studies included in the review using the Mixed Methods Appraisal Tool (MMAT), 2018

Page 19-28: Supplementary 6 (S6): Table 1. Study and participant characteristics (full)

Page 29 – 33: Supplementary 7 (S7): Themes, subthemes and direct participant quote (full)

Page 34 – 39: Supplementary 8 (S8): Quantitative outcomes and direction of findings (full)

Supplementary 1. Detailed database search strings

Search Block	Search Terms <1946 to January 22, 2024> (Embase)	Results
1	Point-of-care systems/ or point-of-care testing/ or rapid diagnostic tests/ or rapid on-site evaluation	33,900
2	(point of care adj3 test*).mp.	29,539
3	(point of care adj3 system*).mp.	5,890
4	bedside test*.mp.	1,671
5	(rapid adj3 test* or evaluation*).mp.	48,862
6	POCT.mp.	4,266
7	POC.mp.	10,692
8	near patient test*.mp.	598
9	1 or 2 or 3 or 4 or 5 or 6 or 7 or 8	87,269
10	(finger* adj3 (prick* or stick* or tip* or puncture* or test*)).mp.	9,750
11	(capillary adj3 (test* or blood suppl*)).mp.	1,656
12	10 or 11	11,323
13	9 and 12	1,081
14	limit 13 to English language	1,070
**15**	limit 14 to yr="2024 -Current" (reviewed noneligible)	132
**Search Block**	**Search Terms** <1806 to January WEEK 3 2024> **(PsycInfo)**	**Results**
2	(point of care adj3 test*).mp.	217
3	(point of care adj3 system*).mp.	275
4	bedside test*.mp.	140
5	(rapid adj3 test* or evaluation*).mp.	1,579
6	POCT.mp.	51
7	POC.mp.	417
8	near patient test*.mp.	4
9	1 or 2 or 3 or 4 or 5 or 6 or 7 or 8	2,489
10	(finger* adj3 (prick* or stick* or tip* or puncture* or test*)).mp.	1,630
11	(capillary adj3 (test* or blood suppl*)).mp.	17
12	10 or 11	1,646

13	9 and 12	32
14	limit 13 to English language	32
**15**	limit 14 to yr="2024 -Current"	0
**Search Block**	**Search Terms** <1806 to January WEEK 3 2024> **(CINAHL)**	**Results**
1	(MH "Point-of-Care Testing+")	5,046
2	"point of care" N2 (test* or system*)	6,981
3	"bedside test*"	356
4	rapid N2 (test* or evaluation*)	4,678
5	POCT	3,558
6	POC	1,481
7	"near patient test*"	3,291
8	S1 OR S2 OR S3 OR S4 OR S5 OR S6 OR S7	12,468
9	finger* N2 (prick* or stick* or tip* or puncture* or test*)	1,322
10	capillary N2 (test* or blood suppl*)	218
11	S9 OR S10	1,524
12	S8 AND S11 Limiters - English Language	131
**13**	S8 AND S11 Limiters – Publication Date: 2024-2025	11

*****Expanders - *Apply equivalent subjects,* Search modes - *Boolean/Phrase (S1 – S12)*

Search Block	**Search Terms** <1946 to January 22, 2024> **(Medline)**	Results
1	Point-of-care systems/ or point-of-care testing/ or rapid diagnostic tests/ or rapid on-site evaluation	20,341
2	(point of care adj3 test*).mp.	13,529
3	(point of care adj3 system*).mp.	17,817
4	bedside test*.mp.	1,084
5	(rapid adj3 test* or evaluation).mp.	28,499
6	POCT.mp.	3,281
7	POC.mp.	8,280
8	near patient test*.mp.	357
9	1 or 2 or 3 or 4 or 5 or 6 or 7 or 8	60,129
10	(finger* adj3 (prick* or stick* or tip* or puncture* or test*)).mp.	5,129
11	(capillary adj3 (test* or blood suppl*)).mp.	1,249
12	10 or 11	6,338
13	9 and 12	588
14	limit 13 to English language	582
**15**	limit 14 to yr="2024 -Current" (reviewed noneligible)	77

Search Block	**Search Terms < January 2024> (Cochrane)**	Results
1	MeSH descriptor: [Point-of-Care Systems) explode all trees	165
2	("point of care" NEAR/2 (test* or system*)):ti,ab,kw	1,469
3	(bedside NEXT test*):ti,ab,kw	69
4	(rapid NEAR/2 (test* or evaluation*)):ti,ab,kw	893
5	(POCT):ti,ab,kw	193
6	(POC):ti,ab,kw	2,739
7	("near patient" NEXT test*):ti,ab,kw	38
8	{OR #1-#7}	4,923
9	(finger* NEAR/2 (prick* or stick* or tip* or test* or puncture*)):ti,ab,kw	1,590
10	(capillary NEAR/2 (test* or blood NEXT suppl*)):ti,ab,kw	1,757

11	{OR #9-#10}	3,209
12	#8 AND #11	78
**13**	#8 AND #11: Date range 01/01/2024 to 01/07/2025 (reviewed noneligible)	2

**Search Block**	**Search Terms < January 2024> (Web of science)**	
1	(("point of care" NEAR/2 test*) OR ("point of care" NEAR/2 system*) OR (rapid NEAR/2 test*) OR "bedside test*" OR "rapid evaluation*" OR "POCT" OR “POC" OR "near patient test*")	
2	AND (finger* NEAR/2 (prick* or stick* or tip* or puncture* or test*)) OR (capillary NEAR/2 (test* or "blood suppl*"))	
3	English language limiters	691
**4**	Publication years: 2024 or 2025 (reviewed noneligible)	69

Supplementary 2. List of excluded studies at full-text screening

**Wrong Outcomes**
POCT devices for urine and no information for finger-prick other than on the advantage of the finger-prick sample
1. Bolodeoku, J., Kuti, M., Melichan, B., Adebisi, S. A Survey of the Awareness, Knowledge, and Experience of Point-of-Care Devices of Doctors in Nigeria. Point of Care: The Journal of Near-Patient Testing & Technology 15(1):p 22-25, March 2016. | DOI: 10.1097/POC.0000000000000084
Not acceptability of finger-prick POCT testing
2. Yonel, Z., Yahyouche, A., Jalal, Z. *et al.* Patient acceptability of targeted risk-based detection of non-communicable diseases in a dental and pharmacy setting. *BMC Public Health* 20, 1576 (2020). https://doi.org/10.1186/s12889-020-09649-7
POCT used but only for venous draws
3. Hayward G, Dixon S, Garland S*, et al* Point-of-care blood tests during home visits by out-of-hours primary care clinicians; a mixed methods evaluation of a service improvement *BMJ Open* 2020;10:e033428. doi: 10.1136/bmjopen-2019-033428
Home self-based capillary testing, only used Net Promotor Score for usability
4. Nwankwo, L., McLaren, K., Donovan, J., Ni, Z., Vidal-Diaz, A., Loebinger, M., Morrisey, A., Igra, A., & Shah, A. (2021). Utilisation of remote capillary blood testing in an outpatient clinic setting to improve shared decision making and patient and clinician experience: a validation and pilot study. *BMJ open quality*, *10*(3), e001192. https://doi.org/10.1136/bmjoq-2020-001192
POCT assessed for eventual patient self-testing at home
5. Joshi, A., Smith, D., Arora, M., & Poston, R. (2008). Anticoagulant monitoring in ventricular assist device patients: a feasibility study. *Interactive cardiovascular and thoracic surgery*, *7*(6), 1035–1038. https://doi.org/10.1510/icvts.2008.186742
Compared capillary sampling modalities: the ear lobe and fingerprick with venepuncture
6. Lightbody, T., Nightingale, P., V Reddy-Kolanu, V., Sapey, E. The Benefits and Limitations of Point of Care Testing for Patients with Diabetes Who Are Acutely Unwell. Journal of Diabetes & Metabolism, 8(9) (2017). https://doi.org/10.4172/2155-6156.1000761.
Fingerprick self-testing at home with venepuncture acceptability
7. Gardiner, C., Williams, K., Mackie, I.J., Machin, S.J. and Cohen, H. (2005), Patient self-testing is a reliable and acceptable alternative to laboratory INR monitoring. British Journal of Haematology, 128: 242-247. https://doi.org/10.1111/j.1365-2141.2004.05300.x
Preference for site capillary POCT was taken – community clinic vs. hospital
8. Shiach, C. R., Campbell, B., Poller, L., Keown, M., & Chauhan, N. (2002). Reliability of point-of-care prothrombin time testing in a community clinic: a randomized crossover comparison with hospital laboratory testing. *British journal*

of haematology, *119*(2), 370–375. https://doi.org/10.1046/j.1365-2141.2002.03888.x
Assessed new lancets for fingerprick testing
9. Ellison T, Hunt S, Henshall S, and Mallya S. 'Effect of Unistik 2 Lancing Device in Reducing Needle-Stick Injuries.' Point of Care 4, no. 1 (2005): 66–68. https://doi.org/10.1097/01.poc.0000160614.05042.ab.
Acceptability based on participation take-up ratios
10. Demorat, H., Lopes, A., Chopin, D., Delcey, V., Clevenbergh, P., Simoneau, G., Evans, J., Mouly, S., Bergmann, J. F., & Sellier, P. (2018). Acceptability and feasibility of HIV testing in general medicine by ELISA or rapid test from finger-stick whole blood. *Presse medicale (Paris, France : 1983)*, *47*(2), e15–e23. https://doi.org/10.1016/j.lpm.2017.11.022
Satisfaction with treatment for diabetes (insulin, tablets or diet)
11. Hirst, J. A., Stevens, R. J., Smith, I., James, T., Gudgin, B. C., & Farmer, A. J. (2017). How can point-of-care HbA1c testing be integrated into UK primary care consultations? - A feasibility study. *Diabetes research and clinical practice*, *130*, 113–120. https://doi.org/10.1016/j.diabres.2017.05.014
**Insufficient data (n = 1)**
<Three questions about pain
12. Muñoz-Chimeno, M., Valencia, J., Rodriguez-Recio, A. *et al.* HCV, HIV AND HBV rapid test diagnosis in non-clinical outreach settings can be as accurate as conventional laboratory tests. *Sci Rep* **13**, 7554 (2023). https://doi.org/10.1038/s41598-023-33925-2
**Wrong population (n= 1)**
Population was >16 years PROSPERO noted >18 years for eligibility
13. Sutcliffe, C. G., Palamountain, K. M., Maunga, S., Searle, K. M., Thuma, P. E., Moss, W. J., & Fisher, M. J. (2018). The feasibility of fingerstick blood collection for point-of-care HIV-1 viral load monitoring in rural Zambia. *Global health innovation*, *1*(2), 3. https://doi.org/10.15641/ghi.v1i2.585
**Publication type (n = 4)**
Poster available only
14. Ferguson C., Ralton L., McCartney E., Dawe J., Richmond J., Tse E., Wigg A., Cock V., Rees T., and Shaw D. Point of Care Testing for Hepatitis C in the Priority Settings of Mental Health, Prisons and Drug and Alcohol Facilities. Journal of Hepatology, The International Liver Congress. London United Kingdom., 77 (2022): S228. https://doi.org/10.1016/S0168-8278%2822%2900828-5.
Abstract available only
15. Herbert S., Carrick G., Edwards S., Copas A., George B., Long L., Sandford C., Amphlett M., and Benn P. Enhancing Services for HIV-Positive Patients: The Role of Point-of-Care CD4 Testing. HIV Medicine, ["17th Annual Conference of the British HIV Association, BHIVA 2011. Bournemouth United Kingdom.", '(var.pagings).'], 12 (2011): 15. https://doi.org/10.1111/j.1468-1293.2011.00925.x

16. Maliszewski K.N., Hsieh Y.-H., Myer D., Perez D.A., Gaydos C.A., Manabe Y.C., Ricketts E., and Rothman R.E. Performance and Patient Acceptability Evaluation of the Chembio DPP HIV-Syphilis Assay in an Emergency Department. Open Forum Infectious Diseases, Infectious Diseases Week, IDWeek 2021. Virtual., 8 (2021): S605. https://doi.org/10.1093/ofid/ofab466.1222
17. Rosset-Zufferey S., Uehlinger D.E., and Sidler D. Capillary point-of-care testing in kidney disease patients. Nephrology Dialysis Transplantation, 59th ERA Congress. Virtual., 37 (2022): i139–i130. https://doi.org/10.1093/ndt/gfac066.095.

Supplementary 3. PRISMA 2020 checklist

Section and Topic	Item #	Checklist item	Location where item is reported
**TITLE**			
Title	1	Identify the report as a systematic review.	Page 1
**ABSTRACT**			
Abstract	2	See the PRISMA 2020 for Abstracts checklist.	Supplementary Dataset 1, S3.
**INTRODUCTION**			
Rationale	3	Describe the rationale for the review in the context of existing knowledge.	Pages 3-4
Objectives	4	Provide an explicit statement of the objective(s) or question(s) the review addresses.	Page 3-4
**METHODS**			
Eligibility criteria	5	Specify the inclusion and exclusion criteria for the review and how studies were grouped for the syntheses.	Page 5
Information sources	6	Specify all databases, registers, websites, organisations, reference lists and other sources searched or consulted to identify studies. Specify the date when each source was last searched or consulted.	Page 6
Search strategy	7	Present the full search strategies for all databases, registers and websites, including any filters and limits used.	Page 6 and Supplementary Dataset 1 S1.
Selection process	8	Specify the methods used to decide whether a study met the inclusion criteria of the review, including how many reviewers screened each record and each report retrieved, whether they worked independently, and if applicable, details of automation tools used in the process.	Page 5-7
Data collection process	9	Specify the methods used to collect data from reports, including how many reviewers collected data from each report, whether they worked independently, any processes for obtaining or confirming data from study investigators, and if applicable, details of automation tools used in the process.	Page 6-7
Data items	10a	List and define all outcomes for which data were sought. Specify whether all results that were compatible with each outcome domain in each study were sought (e.g. for all measures, time points, analyses), and if not, the methods used to decide which results to collect.	Page 5-7 - Table 1. Supplementary Dataset 1. S6;

			S8.
	10b	List and define all other variables for which data were sought (e.g. participant and intervention characteristics, funding sources). Describe any assumptions made about any missing or unclear information.	Page 5-6
Study risk of bias assessment	11	Specify the methods used to assess risk of bias in the included studies, including details of the tool(s) used, how many reviewers assessed each study and whether they worked independently, and if applicable, details of automation tools used in the process.	Page 7 Supplementary Dataset 1 S5.
Effect measures	12	Specify for each outcome the effect measure(s) (e.g. risk ratio, mean difference) used in the synthesis or presentation of results.	Pages 6-7 Table 1. Supplementary Dataset 1. S6; S8.
Synthesis methods	13a	Describe the processes used to decide which studies were eligible for each synthesis (e.g. tabulating the study intervention characteristics and comparing against the planned groups for each synthesis (item #5)).	Pages 6-7 Table 1. Supplementary Dataset 1. S8.
	13b	Describe any methods required to prepare the data for presentation or synthesis, such as handling of missing summary statistics, or data conversions.	Pages 6-7
	13c	Describe any methods used to tabulate or visually display results of individual studies and syntheses.	Pages 6-8 Table 1-2. Supplementary Dataset 1. S6; S8.
	13d	Describe any methods used to synthesize results and provide a rationale for the choice(s). If meta-analysis was performed, describe the model(s), method(s) to identify the presence and extent of statistical heterogeneity, and software package(s) used.	Pages 6-7
	13e	Describe any methods used to explore possible causes of heterogeneity among study results (e.g. subgroup analysis, meta-regression).	N/A
	13f	Describe any sensitivity analyses conducted to assess robustness of the synthesized results.	N/A

Reporting bias assessment	14	Describe any methods used to assess risk of bias due to missing results in a synthesis (arising from reporting biases).	Page 7
Certainty assessment	15	Describe any methods used to assess certainty (or confidence) in the body of evidence for an outcome.	Page 6-7
**RESULTS**			
Study selection	16a	Describe the results of the search and selection process, from the number of records identified in the search to the number of studies included in the review, ideally using a flow diagram.	Page 8-9 Figure 1
	16b	Cite studies that might appear to meet the inclusion criteria, but which were excluded, and explain why they were excluded.	Figure 1 and Supplementary Dataset 1. S2.
Study characteristics	17	Cite each included study and present its characteristics.	Page 8-9, Table 1. Supplementary Dataset 1. S6; S8.
Risk of bias in studies	18	Present assessments of risk of bias for each included study.	Pages 8, Table 1, Supplementary Dataset 1. S6.
Results of individual studies	19	For all outcomes, present, for each study: (a) summary statistics for each group (where appropriate) and (b) an effect estimates and its precision (e.g. confidence/credible interval), ideally using structured tables or plots.	Supplementary Dataset 1. S6
Results of syntheses	20a	For each synthesis, briefly summarise the characteristics and risk of bias among contributing studies.	Page 8, Table 1. Supplementary Dataset 1. S6; S8.
	20b	Present results of all statistical syntheses conducted. If meta-analysis was done, present for each the summary estimate and its precision (e.g. confidence/credible interval) and measures of statistical	N/A

		heterogeneity. If comparing groups, describe the direction of the effect.	
	20c	Present results of all investigations of possible causes of heterogeneity among study results.	N/A
	20d	Present results of all sensitivity analyses conducted to assess the robustness of the synthesized results.	N/A
Reporting biases	21	Present assessments of risk of bias due to missing results (arising from reporting biases) for each synthesis assessed.	N/A
Certainty of evidence	22	Present assessments of certainty (or confidence) in the body of evidence for each outcome assessed.	N/A
**DISCUSSION**			
Discussion	23a	Provide a general interpretation of the results in the context of other evidence.	Pages 21-24
	23b	Discuss any limitations of the evidence included in the review.	Page 23
	23c	Discuss any limitations of the review processes used.	Page 23
	23d	Discuss implications of the results for practice, policy, and future research.	Page 23-24
**OTHER INFORMATION**			
Registration and protocol	24a	Provide registration information for the review, including register name and registration number, or state that the review was not registered.	Page 2
	24b	Indicate where the review protocol can be accessed, or state that a protocol was not prepared.	Page 2
	24c	Describe and explain any amendments to information provided at registration or in the protocol.	Supplementary Dataset 1 – Supplementary 4
Support	25	Describe sources of financial or non-financial support for the review, and the role of the funders or sponsors in the review.	Page 24
Competing interests	26	Declare any competing interests of review authors.	Page 24
Availability of data, code and	27	Report which of the following are publicly available and where they can be found: template data collection forms; data extracted from included studies; data used for all analyses; analytic code; any	All data can be accessed on

other materials		other materials used in the review.	request. Supplementary Dataset 1.

From: Page MJ, McKenzie JE, Bossuyt PM, Boutron I, Hoffmann TC, Mulrow CD, et al. The PRISMA 2020 statement: an updated guideline for reporting systematic reviews. BMJ 2021;372:n71. doi: 10.1136/bmj.n71.

**Section and Topic**	**Item #**	**Abstract Checklist item**	**Reported (Yes/No)**
**TITLE**			
Title	1	Identify the report as a systematic review.	Y
**BACKGROUND**			
Objectives	2	Provide an explicit statement of the main objective(s) or question(s) the review addresses.	Y
**METHODS**			
Eligibility criteria	3	Specify the inclusion and exclusion criteria for the review.	Y
Information sources	4	Specify the information sources (e.g., databases, registers) used to identify studies and the date when each was last searched.	Y
Risk of bias	5	Specify the methods used to assess risk of bias in the included studies.	Y
Synthesis of results	6	Specify the methods used to present and synthesise results.	Y
**RESULTS**			
Included studies	7	Give the total number of included studies and participants and summarise relevant characteristics of studies.	Y
Synthesis of results	8	Present results for main outcomes, preferably indicating the number of included studies and participants for each. If meta-analysis was done, report the summary estimate and confidence/credible interval. If comparing groups, indicate the direction of the effect (i.e. which group is favoured).	Y – as much as space allowed
**DISCUSSION**			
Limitations of evidence	9	Provide a brief summary of the limitations of the evidence included in the review (e.g., study risk of bias, inconsistency and imprecision).	N – space does not

			allow for page 26
Interpretation	10	Provide a general interpretation of the results and important implications.	Y
**OTHER**			
Funding	11	Specify the primary source of funding for the review.	N
Registration	12	Provide the register name and registration number.	Y

From: Page MJ, McKenzie JE, Bossuyt PM, Boutron I, Hoffmann TC, Mulrow CD, et al. The PRISMA 2020 statement: an updated guideline for reporting systematic reviews. BMJ 2021;372:n71. doi: 10.1136/bmj.n71

Supplementary 4. Changes from original PROSPERO protocol (CRD42024512130)

Element changed	Details of change
Anticipated completion date	Changed to accurately reflect inclusion of the systematic review
Inclusion of studies with capillary samples sent off-site	We included one study were finger-prick blood was obtained but not tested on-site and two studies based of hypothetical access to capillary POCT to provide a more comprehensive picture of the literature on the topic.
Inclusion of a different risk of bias tool	We included the MMAT risk of bias tool
Removal of several data categories	We did not report on response rate.

Supplementary 5: Quality assessment of studies included in the review using the Mixed Methods Appraisal Tool (MMAT), 2018

Category of study designs	Methodological quality criteria	Responses			
		Yes	No	Can't tell	Comments
Screening questions (for all types)	S1. Are there clear research questions?				
	S2. Do the collected data allow to address the research questions?				
	Further appraisal may not be feasible or appropriate when the answer is 'No' or 'Can't tell' to one or both screening questions.				
1. Qualitative	1.1. Is the qualitative approach appropriate to answer the research question?				
	1.2. Are the qualitative data collection methods adequate to address the research question?				
	1.3. Are the findings adequately derived from the data?				
	1.4. Is the interpretation of results sufficiently substantiated by data?				
	1.5. Is there coherence between qualitative data sources, collection, analysis and interpretation?				
2. Quantitative randomized controlled trials	2.1. Is randomization appropriately performed?				
	2.2. Are the groups comparable at baseline?				
	2.3. Are there complete outcome data?				
	2.4. Are outcome assessors blinded to the intervention provided?				
	2.5 Did the participants adhere to the assigned intervention?				
3. Quantitative non-randomized	3.1. Are the participants representative of the target population?				
	3.2. Are measurements appropriate regarding both the outcome and intervention (or exposure)?				
	3.3. Are there complete outcome data?				
	3.4. Are the confounders accounted for in the design and analysis?				
	3.5. During the study period, is the intervention administered (or exposure occurred) as intended?				
4. Quantitative descriptive	4.1. Is the sampling strategy relevant to address the research question?				
	4.2. Is the sample representative of the target population?				

	4.3. Are the measurements appropriate?				
	4.4. Is the risk of nonresponse bias low?				
	4.5. Is the statistical analysis appropriate to answer the research question?				
5. Mixed methods	5.1. Is there an adequate rationale for using a mixed methods design to address the research question?				
	5.2. Are the different components of the study effectively integrated to answer the research question?				
	5.3. Are the outputs of the integration of qualitative and quantitative components adequately interpreted?				
	5.4. Are divergences and inconsistencies between quantitative and qualitative results adequately addressed?				
	5.5. Do the different components of the study adhere to the quality criteria of each tradition of the methods involved?				

From: Hong QN, Pluye P, Fàbregues S, Bartlett G, Boardman F, Cargo M, Dagenais P, Gagnon M-P, Griffiths F, Nicolau B, O'Cathain A, Rousseau M-C, Vedel I. Mixed Methods Appraisal Tool (MMAT), version 2018. Registration of Copyright (#1148552), Canadian Intellectual Property Office, Industry Canada.

Supplementary 5.1: Quality assessment of studies included in the review using the Mixed Methods Appraisal Tool (MMAT), 2018. FULL.

				1. Qualitative					2. Randomized Controlled Trial					3. Quantitative non-randomized					4. Quantitative Descriptive					5. Mixed methods					MMAT Scoring
S. No.	**Author (year)**	S1	S2	1.1	1.2	1.3	1.4	1.5	2.1	2.2	2.3	2.4	2.5	3.1	3.2	3.3	3.4	3.5	4.1	4.2	4.3	4.4	4.5	5.1	5.2	5.3	5.4	5.5	
1.	Bajis et al (2018)[20]	Y	Y																Y	Y	N	C	Y						●●●○○
2.	Bogers et al (2015)[41]	Y	Y						C	Y	Y	N	Y																●●●○○
3.	Kelly et al (2021)[23]	Y	Y																Y	Y	Y	Y	Y						●●●●●
4.	Kamhi-Nesher et al (2022)[24]	Y	Y																Y	Y	Y	Y	Y						●●●●●
5.	Nielsen et al (2012)[26]	Y	Y						C	Y	Y	N	Y																●●●○○
6.	Atkins et al (2022)[22]	Y	Y																Y	Y	Y	Y	Y						●●●●●
7.	Woods et al (2004)[27]	Y	Y						Y	Y	Y	Y	Y																●●●●●
8.	Butler et al (2020)[13]	Y	Y	Y	Y	Y	Y	Y						Y	Y	Y	Y	Y						Y	Y	Y	Y	Y	●●●●●
9.	Butler et al (2021)[40]	Y	Y	Y	Y	Y	Y	Y																					●●●●●
10.	Riva et al (2020)[43]	Y	Y											Y	Y	Y	Y	Y											●●●●●
11.	Yonel et al (2020)[42]	Y	Y											Y	Y	Y	Y	Y											●●●●●
12.	Smits et al (2022)[21]	Y	Y											Y	Y	Y	N	Y											●●●●○
13.	Laurence et al (2010)[16]	Y	Y						Y	Y	Y	N	Y																●●●●○
14.	Daneau et al (2016)[48]	Y	Y											Y	C	Y	N	Y											●●●○○

15.	Conway et al (2015)[49]	Y	Y																Y	Y	Y	C	Y						●●●●○
16.	Glover et al (2008)[46]	Y	Y	Y	Y	Y	Y	Y																					●●●●●
17.	Butler et al (2008)[50]	Y	Y	Y	Y	Y	Y	Y																					●●●●●
18.	Gauthier et al (2012)[47]	Y	Y																Y	Y	C	Y	Y						●●●●○
19.	Herbert et al (2012)[45]	Y	Y																Y	Y	Y	Y	C						●●●●○
20.	Liu et al (2003)[44]	Y	Y																Y	Y	N	Y	Y						●●●●○
21.	Pourafshar et al (2024)[28]	Y	Y																Y	Y	Y	Y	Y						●●●●●

Abbreviations: Y = Yes; N = No; C = Can't tell. **Notes:** Rating: Low risk = <3●●●○○; Medium risk = <4●●●●○; High risk = 5●●●●●
Butler et al (2020)[7] score on quantitative description section 1.4 was scored based on 'complete data on cases' instead of nonresponse bias as this was not stated explicitly.

Supplementary 6. Table 1. Study & Participant Characteristics. (FULL)

					Participants			
Author (Year); Country; Setting/service	Study Purpose	Parameters; POCT device	Sample Size[a]	MMAT scoring	Age (range)	Male (n)	Female (n)	Relevant findings
**Bajis et al. (2018) [20] Australia**<br><br>Outpatient clinics	Acceptability of finger-stick versus venepuncture HCV RNA testing.	Hepatitis C virus.<br><br>Xpert HCV	565 participants	Low<br>●●●○○	44 years (18->56)	387<br><br>Transgender (n)<br>6	172	Finger-stick testing preferred (65%) for quickness (62%) and venous (35%) access issues (21%). Female gender and recent injecting drug use were associated with significantly greater odds of preferring finger-stick over venepuncture.
**Bogers et al. (2015) [41] Netherlands**<br><br>Mental health outpatient centres and inpatient wards.	Preferences between traditional venous sampling and capillary (finger-prick) POCT.	WBC & ANC<br><br>HemoCue WBC DIFF	70 patients<br>23 clinicians	Low<br>●●●○○	42.4 years (42-49)	53	17	Strong preference for finger-prick sampling (mean 7.9/10; 0 = venous preferred, 10 = capillary preferred); inpatients rated it higher than outpatients. Finger-prick was rated as significantly less painful, worrying, and burdensome than venous. Sampling method had a moderate

								influence on clozapine motivation (mean 4.0/10; 0 = not at all, 10 = very much). Clinicians preferred capillary (mean 8.9/10); it also reduced time and improved patient cooperation.
**Kelly et al. (2021) [23] Australia**<br><br>Mental health outpatient centres and inpatient wards	Feasibility and satisfaction of finger-prick POCT versus venous sampling for ANC monitoring.	WBC & ANC<br><br>AthelasOne	20 patients<br><br>20 clinicians	High<br>● ● ● ● ●	35.7 years	15	5	60% of patients preferred POC fingerstick. >75% reported benefits including immediate results, in-office testing, and no lab handling. Trend toward greater worry with venous draws (p = .0685). Clinicians consistently preferred POC, citing ease of use, improved care coordination, and potential to support clozapine uptake and adherence.
**Kamhi-Nesher et al. (2022) [24] Israel**	Feasibility of POCT from both patient and clinician perspectives.	Clozapine levels<br><br>MyCare Insite	Patient: 44<br>Clinician: 6	High<br>● ● ● ● ●	43 years (18-71)	27	17	Over 60% valued TDM; 49% preferred frequent testing. Capillary sampling favoured

Mental Health outpatient centre and inpatients wards.								by 58% of patients and 81% of clinicians. 11% said sampling method would affect adherence. Most (72%) preferred in-office testing over lab-based sampling.
**Nielsen et al. (2012) [26] Denmark**<br><br>Mental Health outpatient clinic.	Tolerability of capillary (finger-prick) POCT compared to traditional venous blood testing.	WBC and granulocytes<br><br>Chempaq Express Blood Counter (Chempaq XBC)	Patient: 77<br><br>Clinician: 74	Low<br>●●●○○	45.6 years	51	34	Capillary blood sampling was preferred by 63% of patients; reported as less painful and more convenient ($p < 0.001$). Nearly half (47%) were unaware of the reason for mandatory blood testing. 87% of clinicians found POCT more convenient for their patients.
**Atkins et al. (2022) [22] UK**<br><br>Mental health outpatient clinic.	Acceptability of POCT among patients with SCZ and clinicians.	Clozapine levels<br><br>MyCare Insite	Patient: 106<br><br>Clinician: 10	High<br>●●●●●	47 years (21-75)	55	29	Capillary testing was acceptable to 98% of patients, with 85% preferring it to venous sampling. 78% valued immediate results, 90% saw improved care, and 83% felt more

								involved. All clinicians (100%) rated the method positively and easier than venous collection.
**Woods et al. (2004) [27] Canada**<br><br>Anticoagulation outpatient clinic	Satisfaction with conventional venous INR testing versus capillary (finger-prick) POC INR testing.	INR<br><br>CoaguChek-S	Patient: 60	High<br>● ● ● ● ●	64.4 years (capillary cohort)<br><br>59.5 years (venous cohort)	28	32	Capillary INR testing was significantly preferred (VAS: 1.64 vs 4.45; 0 = very satisfied, 10 = very dissatisfied; $p < 0.001$) and less painful (VAS: 0.83 vs 2.23; 0 = no pain, 10 = very painful; $p = 0.004$) compared to venous testing. Turnaround time was also shorter (42 vs 75 minutes; $p < 0.001$).
**Butler et al. (2020) [13][c] UK**<br><br>Community mental health centre	The impact of POCT on the completion of physical health checks in mental health settings.	Lipid panel & HBA1c<br><br>Afinion 2	Patient: 14<br><br>Clinician: 15	High<br>● ● ● ● ●	33 years (19-66)	10	4	POC testing increased test completion in EIS teams (RRs >2.6; $p < 0.001$), but effects waned after adjusting for historical trends. Clinicians noted barriers including unclear roles, workload, and

								device issues, though confidence improved with use. Patients reported reduced anxiety, greater comfort, and valued real-time results for personalised care and better understanding of physical health.
**Butler et al. (2021) [40]**[b]<br><br>Community mental health team	Factors influencing engagement with and implementation of POCT in clinical settings.	Lipid panel & HbA1c<br><br>Afinion 2	Clinician: 15	High<br>● ● ● ● ●	ND	7	8	Initial engagement with POCT varied due to anxiety, workload, and perceived value. Usability improved with experience. Clinicians valued POCT for enhancing autonomy, efficiency, and patient access, though some noted device issues and concerns about professionalism during errors.
**Riva et al. (2020) [43] Malta**	Satisfaction with POCT versus laboratory testing.	INR<br><br>CoaguChek XS Plus	Patient: 313	High<br>● ● ● ● ●	75 years (65-78) (POC cohort)	165	148	Patients in the POCT INR group reported significantly higher satisfaction than those in the

Anticoagulation outpatient health centres					70.5 years (63-76) (Lab cohort)			laboratory cohort across the subscales of hassles/burden, psychological effect, and convenience, as well as overall anticoagulant treatment satisfaction.
**Yonel et al. (2022) [42] UK**<br><br>Hospital outpatient centre	Acceptability of POCT compared to laboratory testing.	HbA1c, Creatinine, & Vitamin D<br><br>Nova StatSensor (creatinine/eGFR); Siemens DCA-Vantage (HbA1c); and CityAssays (vitamin D)	Patient: 158	High<br>● ● ● ● ●	ND	ND	ND	Most participants reported significantly less discomfort with POCT than with venepuncture, despite similar levels of residual pain across both groups.
**Smits et al. (2022) [21] Netherlands**<br><br>General practices	Explore patient and clinicians' experiences of using POCT for diabetes management in general practice.	HbA1c & Glucose<br><br>Afinion 2<br><br>AccuChek Inform II	Patient: 104<br><br>Clinician: 65	Medium<br>● ● ● ● ○	64.6 years (34-81) HbA1c<br><br>59.5 years (21-79) Glucose	52	52	Patients and clinicians reported high satisfaction with POCT and willingness to continue its use, though additional workload from quality assurance procedures was noted as a barrier.
**Laurence et al. (2010) [16] Australia**	Satisfaction with POCT versus laboratory testing	Lipid panel, HbA1c & INR<br><br>DCA 2000	Patient: 4968	Medium<br>● ● ● ● ○	67 years (59-75)	2648	2320	POCT patients reported higher overall satisfaction, citing convenience and improved

General practices		CoaguChek S Cholestech LDX.						disease management. Finger-prick sampling and immediate feedback enabling real-time discussion were rated significantly more favourable than venous sampling.
**Daneau et al. (2016) [48] South Africa and Belgium**<br><br>Outpatient clinics	Assess patient attitudes toward finger-stick POCT	CD4, Haemoglobin, ALT and Creatinine.<br><br>Pima CD4 Analyser	Patient: 499	Low ● ● ● ○ ○	46 years (19-83)	351	148	More patients preferred finger-stick over venepuncture (38.5% vs 25.5%), while 36.5% had no preference. Finger-stick was viewed as easier, especially for those with difficult vein access. Clinicians more often favoured venepuncture due to concerns about exposure to infectious blood.
**Conway et al. (2015) [49] Australia**<br><br>Sexual health outpatient clinics	Acceptability of rapid HIV finger-prick testing.	CD4 & viral load / HIV<br><br>Determine HIV Combo Assay	Patient: 1093	Medium ● ● ● ● ○	25-34 years (18 - >45)	1093	-	59% reported less stress and anxiety with rapid finger-prick testing compared to conventional sampling. Overall,

								satisfaction was high (98%), with 79% preferring it over conventional testing, 93% willing to recommend it, and 50.3% willing to pay for it.
**Glover et al. (2008) [46][b] Australia**<br><br>Hospital	Health professionals' perception of POCT for warfarin management.	INR<br><br>Hypothetical finger-prick POCT	Clinician: 33	High<br>● ● ● ● ●	34-55 years	11	22	Perceived benefits of POCT included cost-effectiveness, time efficiency, and enhanced patient experience; barriers included limited knowledge, low operator confidence, and accreditation requirements.
**Butler et al. (2008) [50][b] UK**<br><br>General practices	Clinicians' views on POCT for common infections	Viral and bacterial viruses<br><br>Hypothetical finger-prick POCT	Clinician: 40	High<br>● ● ● ● ●	ND	ND	ND	Clinicians expressed strong enthusiasm for finger-prick POCT to distinguish viral from bacterial infections, though concerns were noted regarding time constraints, device maintenance, quality control, and cost.
**Gauthier et al. (2012) [47] France**	Assess feasibility and acceptability of finger-prick RHT	CD4 & viral load<br><br>RHT VIKIA HIV ½ (BioMerieux)	Patient: 383<br><br>Clinician: 72	Medium<br>● ● ● ● ○	36.2 years (19-70>)<br><br>48.7 years*	196<br><br>38*	187<br><br>34*	Most patients (96.9%) preferred receiving results during the consultation, and

Community healthcare centres								over 80% reported finger-prick RHT as less painful than venous testing. Over 55% of clinicians were satisfied and intended to continue its use.
**Herbert et al. (2012) [45] UK**<br><br>HIV outpatient centres	Evaluate patient acceptability of the PIMA POC CD4 test.	CD4<br><br>PIMA CD4 Analyser	Patient: 283	Medium ● ● ● ● ○	41 years (21-66)	246	37	54% of patients preferred POCT over laboratory testing, with 87% willing to wait 20 minutes for results; preference was significantly associated with higher CD4 counts.
**Liu et al. (2003) [44] Thailand**<br><br>Family outpatient planning clinics and inpatient postpartum wards	Acceptability of rapid finger-prick testing.	HIV antibodies<br><br>Hema Strip HIV-1/2	Patient: 804	Medium ● ● ● ● ○	27 years (16-45)	ND	804	Most patients were satisfied (97%) and 79% preferred finger-prick testing over venepuncture.
**Pourafshar et al. (2024) [28] USA**<br><br>Ancillary healthcare community site.	Experiences, satisfaction, and preference for finger-prick capillary blood testing	Haemoglobin & Haematocrit<br><br>BD MiniDraw	Patient: 113	High ● ● ● ● ●	49 years (18-71)	33	74	90% of patients reported a positive experience with finger-prick testing, and 84% preferred it over venous sampling, citing site convenience, time

								savings, and staff professionalism.

Note: UK = United Kingdom; USA = United States of America; WBC = White blood counts; ANC = Absolute neutrophil counts; TDM = Therapeutic drug monitoring; INR = International normalised ratio; CD4 = Clusters of differentiation 4; HBA1C = Hemoglobin; HCT = Hematocrit; HIV= Human immunodeficiency virus; RHT = Rapid human immunodeficiency; ALT = Alanine aminotransferase; GP = General practitioners; Not applicable = -; ND = Not documented. Age and gender of clinical staff documented where reported.
[a]Based on total sample.
[b]Qualitative.
[c]Mixed-Methods.

Supplementary 7. Themes, subthemes and direct participant quotes [FULL]

Themes	Subthemes	Source	Participant Type	Supporting Quotes
**Theme 1:** Enhancing the patient-clinician relationship through finger-prick point-of-care testing: perceptions of engagement, communication, and care experience	**Subtheme 1.1:** Improved care, communication, and understanding of health management and treatments	Atkins et al., 2022	Patients	"Helped with my understanding," "better results, clearer understanding," "I know more about what I'm having"<br>"Better to know results straightaway," "advice on the spot," "find out straight away and what to do next"<br>"Useful to know, as when I receive a text with my results, it's just numbers and I can't ask anything
		Atkins et al., 2022	Clinicians	"Facilitated faster changes to prescription and allowed the patient to be informed on their clozapine levels at the time of consultation," "Medication changes discussed face-to-face," and "doctors informed straight away"<br>"There is also no need to chase results from the laboratory after the clinic"
		Butler et al., 2020	Patient	"It's handy because all the questions that I had in mind I could ask then and get a professional opinion on it, whereas if they sent a text, I would look at some numbers and I wouldn't really register it"
		Butler et al., 2021	Clinician	"She had been in the team for a long time, and she would never want to get her bloods done [via venous], she always refused and then she said OK I will have it done, and so for her to actually change her attitude was brilliant"

		Butler et al., 2008	Clinician	"We could explain to the patient we have done the test and it's a viral thing, and antibiotics are not going to work, which is justified by the results, as patients want the evidence as well"
	**Subtheme 1.2:** Perceived Experience, Preferences, and Tolerability of Finger-Prick POCT	Nielsen et al., 2021	Patients	"The capillary blood test is less painful," "I prefer regular blood sampling because that is what I am used to"
		Atkins et al., 2022	Clinicians	"Much easier to obtain a sample from patients with small or obscure veins," "I like that it is easier and simpler than taking [venous] bloods." "Able to take immediate action or escalate if required," "Quicker results and can make changes to medication"
		Butler et al., 2021	Clinicians	"It was easier to then put their responses to the questions together with the blood results and then the patient could see how their diet and lifestyle choices could be impacting on the results they got from the blood," "It's not just nurses now, the occupational therapists (OTs) and social workers are doing [physical health] clinics too because it's accessible to everyone"
		Butler et al., 2008	Clinicians	"Oh my God, it would be like Christmas. It would make my day," "Yes, if it was available, then yes. Then at least we can explain to the patient that we have done the test, it is a viral thing, and the antibiotics are not going to work for this condition"
	**Subtheme 1.3:** Psychological impact and perceived convenience of finger-prick testing	Bogers et al., 2015	Patients	[Venous testing] "Causes more anxiety", "My blood is used for different purposes, or it is sold for money," [Capillary testing] "Is convenient, can be done anywhere, by your own nurse", "When I was psychotic capillary sampling would have helped me a lot"

		Nielsen et al., 2021	Patients	“I don't have to wait at the laboratory” “I get the test values right away, which makes me more comfortable. I used to get worried waiting for the results”<br>“I don't have to face all the people at the laboratory” “This new blood sampling is drawn by my primary care person, which makes me more comfortable”
		Glover et al., 2008	Clinician	‘It’d be great for patient advocacy and empowering them to take some responsibility for their own health care”
		Butler et al., 2020	Patient	“More anxious going in than coming out” – “It makes me feel more secure. I’m less worried now and I don’t feel that I need another health check for a while”<br>“I turned [the GP] down because of the anxiety of going, so this was [finger-prick] the only way really I could have the test”<br>“Because like usually when the GP gets my blood tests done, they like have to bring other nurses in to do it because they can’t find my vein properly”
**Theme 2:** Clinical implications of finger-prick POCT on clinician workflow, confidence, and skill acquisition.	**Subtheme 2.1:** Finger-prick POCT errors, accuracy, and maintenance.	Butler et al., 2021	Clinicians	“One would think that the accuracy is probably not as fabulous as sending it to the lab,” “I didn’t know how to correct what I’d done wrong, so I didn’t want to put them through the same situation again”- “It’s getting the sequence … which is not very easy. And it looks bad in front of the patient”
		Butler et al., 2020	Clinicians	“We gave up doing the lipids in the end because every time we had problems with it unless you guys [POCT researchers] were there,”

				"There were too many errors that often would leave the patient waiting, then just have to say, you know, we couldn't leave them waiting that long. So, we didn't get on so well with it," "It just seemed a little bit unprofessional when I was in somebody's house that was quite willing to participate, and then I couldn't complete the whole thing"
	**Subtheme 2.2:** POCT Training and skill requirements, workload constraints, and sample turnaround time	Butler et al., 2021	Clinicians	"The more skills and the wider they are, should give that service user more confidence in that," "It's important for me to be able to do this myself, and that that would give me responsibility; it would also ensure that in all areas of what they need I can provide for that. So, it was being a more complete practitioner that was really the driver for me"<br>"I did do the training for having a blood test … but I found the opportunities to practise doing it were quite few and far between."<br>"Because of the amount of time that I will spend chasing results, I'll just go and do it myself [on the POC device] and get the results there and then. So, it's making my work a lot easier."<br>"The more you do it the more you kind of come across the different things that will happen and the more confident you are in just reassuring them but also still getting a result"
		Butler et al., 2008	Clinician	"I can imagine there will be a lot of implications in maintaining this equipment with calibration and all the things that go with maintaining near-patient testing

		Glover et al., 2008	Clinician	“What we need is nurses to be able to do this and then nurse prescribers … I think having a nurse prescriber [is important] for continuity of journey and best practice”<br>“We’ve had no research presented to us”

Supplementary 8. Quantitative acceptability findings from the 17 studies reporting extractable quantitative outcome measures (FULL)

Study	Population / Setting	Outcome Type	Measure of Acceptability	Key Quantitative Findings	Overall Direction of Findings
Bajis et al. (2018)	Patients<br><br>Outpatient setting	Proportion and Likert-scale (%)	Acceptability and preference	83% rated finger-prick as very acceptable vs 72% for venepuncture. 65% favoured finger-prick vs 35% venepuncture.	Favours finger-prick POCT
Bogers et al. (2015)	Patients and clinicians<br><br>Mixed inpatient/outpatient setting	VAS (mean scores)	Preference and burden	Mean preference score favoured finger-prick testing (7.9/10). Lower burden reported with finger-prick sampling. Clinicians also favoured finger-prick POCT (mean 8.9/10).	Favours finger-prick POCT
Kelly et al. (2021)	Patients and clinicians<br><br>Mixed inpatient/outpatient setting	Proportion (%) and Likert-scale	Preference and satisfaction	60% of patients favoured finger-prick testing. >75% of patients reported perceived benefits of immediate results, eliminating unknown people touching samples, not sending blood offsite, and immediate access to results improved treatment. Clinicians consistently rated POCT as preferable.	Favours finger-prick POCT
Kamhi-Nesher et al (2022)	Patients and clinicians	Proportion (%)	Preference and test feasibility	58% of patients and 81% of clinicians favoured finger-prick POCT. 72%	Favours finger-prick POCT

	Mixed inpatient/outpatient setting			preferred testing in clinicians' office than a phlebotomy laboratory.	
Nielsen et al. (2012)	Patients and clinicians<br><br>Outpatient setting	Proportion (%) and VAS	Preference, pain, convenience	62.8% favoured finger-prick POCT, 11.6% venous testing, 25.6% no preference. Lower perceived pain (0.55 cm 25–75 percentiles: 0.1–1.4 vs. 1.75 cm 25–75 percentiles: 0.7–2.6, p< 0.001) and inconvenience with finger-prick testing (0.3 cm 25–75 percentiles: 0.05–0.7 vs. 2.3 cm 25–75 percentiles: 0.75–4.5, p < 0.001). 87.1% clinicians reported greater perceived convenience.	Favours finger-prick POCT
Atkins et al. (2022)	Patients and clinicians<br><br>Outpatient setting	Proportion (%)	Preference and experience	77% (aripiprazole) and 88% (clozapine) favoured finger-prick testing. 100% (aripiprazole) and 88% (clozapine) felt the test provided them with better care. High satisfaction reported. 100% clinicians preferred POCT.	Favours finger-prick POCT
Woods et al. (2004)	Patients<br><br>Outpatient setting	VAS (mean scores)	Preference and pain	Lower VAS scores (0 = very satisfied) for finger-prick testing (1.64 vs	Favours finger-prick POCT

				4.45 venous). Perceived pain (0 = no pain) was lower with finger-prick (0.83 vs 2.23 venous).	
Riva et al. (2020)	Patients<br><br>Outpatient setting	Mean scores (DASS and PACT-Q2 scales)	Satisfaction, burden, convenience	Mean overall DASS scores were lower in the POCT group (50.8 vs 59.4, p<0.001), indicating higher satisfaction and lower burden. POCT users also reported greater convenience (PACT-Q2 convenience score 85.9 vs 79.8, p<0.001) and higher treatment satisfaction (67.6 vs 65.2, p=0.039).	Favours finger-prick POCT
Yonel et al. (2022)	Patients<br><br>Outpatient setting	VAS (median scores)	Pain	Median pain scores were lower for finger-prick POCT compared with venous sampling (7/100 vs 17/100). Participants reported lower discomfort. Finger-prick testing was identified as acceptable and was generally perceived as less uncomfortable than venepuncture.	Favours finger-prick POCT
Smits et al. (2022)	Patients and clinicians<br><br>General practitioners	Proportion (%) and NPS	Satisfaction and acceptability	87-100% of patients reported positive experiences. Most patients considered POCT convenient and	Non-comparative findings

				valued immediate results. The majority of clinicians supported continuation of finger-prick POCT and reported positive experiences relating to workflow integration and patient engagement.	
Laurence et al., (2010)	Patients<br><br>General practitioners	Likert scale	Preference, convenience, satisfaction	Compared with laboratory testing, patients receiving finger-prick POCT reported greater satisfaction with the collection process (7.8 vs 5.1, p<0.001), convenience (8.9 vs 8.7, p=0.009), and disease management, including improved understanding and motivation relating to their condition (9.0 vs 8.7, p=0.003; 8.9 vs 8.2, p<0.001). Patients also reported that POCT strengthened their relationship with their GP (8.3 vs 8.1, p=0.010). Confidence in the information provided by POCT remained high (9.0 vs 8.9, p=0.010).	Favours finger-prick POCT
Daneau et al. (2016)	Patients and clinicians<br><br>Outpatient setting	Proportion (%)	Preference and experience	66% of participants preferred one finger-prick over venepuncture, while 35% preferred two	Mixed findings

				finger-pricks and none preferred venous sampling. Following multiple finger-pricks, preference declined, with 54% preferring three finger-pricks before venepuncture and 4.3% preferring venous sampling. Clinicians reported mixed views relating to repeated finger-prick sampling and workflow practicality.	
Conway et al. (2015)	Patients<br><br>Outpatient setting	Proportion (%)	Preference, comfort, satisfaction	78.8% preferred finger-prick testing. 60.7% found it more comfortable. High satisfaction (98%); 21.5% concerned about reliability.	Favours finger-prick POCT
Gauthier et al. (2012)	Patients and clinicians<br><br>Mixed inpatient/outpatient setting	Proportion (%) and VAS	Acceptability, pain, satisfaction	99.7% acceptability; 93.8% would recommend. >80% reported less pain with finger-prick than normal sampling. Clinician satisfaction scores are moderate (61%)	Non-comparative findings
Herbert et al. (2011)	Patients<br><br>Outpatient setting	Proportion (%) and Likert-scale	Acceptability, preference, comfort	High acceptability (97%). 57% preferred finger-prick POCT. Majority did not find it more uncomfortable than venous testing.	Favours finger-prick POCT

Liu et al. (2023)	Patients<br><br>Mixed inpatient/outpatient setting	Proportion (%)	Preference and satisfaction	56.2% preferred finger-prick sampling. 78.7% preferred rapid POC testing. 96.8% were satisfied.	Favours finger-prick POCT
Pourafshar et al. (2024)	Patients<br><br>Outpatient setting	Proportion (%)	Preference and satisfaction	65% preferred finger-prick vs 15% venous. High satisfaction and 83% willing to use finger-prick testing again.	Favours finger-prick POCT

Note: Direction of findings reflects overall interpretation of reported quantitative acceptability outcomes and should be interpreted alongside narrative synthesis. This table include studies reporting extractable quantitative outcomes relating to acceptability, preference, satisfaction, burden, convenience, or perceived experience of finger-prick POCT. Four included studies (three qualitative studies and one mixed-methods study) did not report relevant quantitative acceptability outcomes and are therefore not included in this table. DASS = Duke Anticoagulation Treatment Questionnaire; PACT-Q2 = Perception of Anticoagulation Treatment Questionnaire.
