# Supplementary material for: Acceptability of capillary point-of-care testing: a systematic review
Source: BMJ Open. 2026 Sep 15;16(9):e115234. doi: 10.1136/bmjopen-2025-115234 (PMC13583689; doi:10.1136/bmjopen-2025-115234)
Supplement: Supplementary file 2 [file bmjopen-2025-115234.reviewer_comments.pdf]

PEER REVIEW HISTORY

BMJ Open publishes all reviews undertaken for accepted manuscripts. Reviewers are asked to complete a checklist review form and are provided with free text boxes to elaborate on their assessment. These free text comments are reproduced below.

ARTICLE DETAILS

Title (Provisional)

Acceptability of Capillary Point-of-Care Testing: A Systematic Review.

Authors

Dunnett, Danielle April; Casetta, Cecilia; Oloyede, Ebenezer; Loane, Emilia; MacCabe, James; Taylor, David

---

VERSION 1 - REVIEW

**Reviewer**	**1**
**Name**	**Markwart, Robby**
**Affiliation**	**Jena University Hospital, Institute of General Practice and Family Medicine**
**Date**	**07-Jan-2026**
**COI**	**None**

The study entitled "Acceptability of Capillary Point-of-Care Testing: A Systematic Review" synthesizes evidence from published studies on satisfaction, experiences, acceptability, and preferences related to finger-prick versus venous point-of-care testing. Based on their findings, the authors conclude that finger-prick POCT is more acceptable to both patients and clinicians, and is less distressing and more convenient than venipuncture.

Comments:

• The topic of the acceptability of point-of-care testing is both interesting and highly relevant, as patient experiences and preferences are one of the three cornerstones of evidence-based medicine.

• Overall, the finding that both patients and clinicians largely prefer finger-prick sampling over venous sampling is not surprising or groundbreaking. However, the finding that finger-prick point-of-care testing can enhance the patient–clinician relationship through improved engagement, communication, and understanding of care is highly relevant.

• Based on the inclusion criteria, the study only included articles that assessed satisfaction, experience, acceptability, or preferences in direct comparisons between finger-prick and venous sampling. As a result, many studies on the acceptability of finger-prick point-of-care

testing were excluded because they did not include a comparison with venous sampling. This represents an important limitation of the study and should be explicitly acknowledged in the title, abstract, and conclusions. For example, there are numerous studies on finger-prick C-reactive protein POCT and other biomarkers that have examined acceptability, experiences, and satisfaction among both patients and clinicians. However, the vast majority of these studies were not included because they did not provide a direct comparison with venous sampling. In my view, it is problematic to draw general conclusions about finger-prick POCT when a substantial body of relevant evidence is excluded. Studies focusing exclusively on finger-prick POCT may reveal additional or different findings regarding acceptability, preferences and experiences. I therefore strongly recommend that the authors revise the manuscript to clearly reflect this restriction and limit their conclusions to comparisons between finger-prick and venous sampling.

• The authors should explain how the search strings were developed, validated, and tested. Notably, almost 30% of the included studies (6 out of 21) were not identified through the search strategy but were instead retrieved via other methods, such as manual hand searching and citation tracking. This raises concerns regarding the sensitivity of the search strategy.

• Figure 1: The figure misses the number of excluded studies after abstract screening.

• In the abstract, the authors state that “Medline, Embase, PsycINFO, CINAHL, Cochrane, and Web of Science were searched from inception to July 2025.” However, the detailed database search strings provided in the Supplementary Material indicate that the searches were conducted in January 2024. The authors should clearly and consistently report the timing of the database searches in both the abstract and the Methods section.

**Reviewer**	**2**
**Name**	**Plebani, Mario**
**Affiliation**	**Università di Padova, Medicine-DIMED**
**Date**	**12-Jan-2026**
**COI**	**None**

The authors do not seem to have enough knowledge on the issue of POCT and some statements (e.g the reduction of TAT and improved effectiveness) are not evidence-based and references not appropriated.

The question about capillary versus venous sampling is not limited to POCT and in general it requires much more and deeper concern

---

**Reviewer**	**3**
**Name**	**Singh, Neha**

**Affiliation**	**AIIMS Patna, Transfusion Medicine**
**Date**	**19-Jan-2026**
**COI**	**None**

It was a pleasure to review the manuscript, acceptability of Capillary Point of Care Testing: a systematic Review.

I would like to add additional comments, as authors need to address more studies that showed limitations. Kindly address the following points;

1. Higher User-Dependent Variability studies like: 1. Operator Inexperience: Unlike laboratory technicians, POCT is often performed by untrained or multi-tasking staff, increasing the risk of errors in the pre-analytical phase (e.g., inadequate sample volume, poor technique).

2. studied related to Procedural Inconsistency:

3. Studies of blood donation screening where point-of-care testing is primarily used at the initial stage.

4. Studies which are showing limitations of point of care compare quality standards testing.

**Reviewer**	**4**
**Name**	**van der Pol, Barbara**
**Affiliation**	**Indiana University, x**
**Date**	**06-Feb-2026**
**COI**	**None**

This is one of the best reviews, on any topic, that I have critiqued! I have no comments or suggestions

---

**Reviewer**	**5**
**Name**	**Honarmand, Kimia**
**Affiliation**	**London Health Sciences Centre, Medicine**
**Date**	**09-Mar-2026**
**COI**	**None**

Thank you for the opportunity to review this manuscript, describing a systematic review of the literature on the acceptability of POCT among patients and clinicians. The broad scope of this review, spanning across care settings, is certainly unique and interesting. The study uses appropriate systematic review methodology.

I have some comments for the authors' consideration:

Major comments:

- The authors mention that they synthesized quantitative data as well, but this is not apparent in the Results section. The absence of quantitative data pooling is notable. Some individual study quantitative data are described, but the data were not pooled. What was the reason the authors focused solely on studies that reported qualitative findings? What about studies that reported proportion of patients and clinicians who found POCT acceptable and to what degree? Were these studies excluded unless they reported qualitative data? If so, what was the rationale for this? There could have been an opportunity to pool quantitative data regarding acceptability across studies and perhaps even compare across care settings.

- What was the rationale for limited the search to English language articles? With the technology now available to support translations, there is a push towards SRMAs not being limited to English language articles.

- Introduction: "*Nonetheless, concerns remain regarding analyser accuracy(30),*

regulatory compliance(31), and the need for regular calibration and quality control(32). Prior

reviews have often been limited to specific professional groups(32), settings(33), or

conditions(15, 34)". I believe this is the rationale statement to justify this study, but does not seem to match the intent of the systematic review. This review was specifically focused on acceptability among patients and clinicians. I would suggest keeping the preceding statement as the points still stand, and adding a subsequent statement to describe the importance of patient and clinician acceptability (in addition to those other factors listed).

- Please mention that inpatient and outpatient settings were included in the Methods section. And please specify the number of studies that focused on inpatient use of POCT vs. outpatient. This will have important implications for readers.

- Implications and Conclusions: "*Finger-prick POCT is not only acceptable but consistently preferred over venous sampling."* The authors have acknowledged that the acceptability of POCT is not consistent. Please revise this statement to reflect the findings (ie, most find it acceptable/ prefer it, but not all).

Minor comments:

- Results: "Psychological affect and convenience of sampling methods". I believe the authors are referring to psychological effects (ie, impacts)?

- Same subheading as above: The term "Convenience of sampling method" is a bit unclear. Is this referring to the perceived convenience of POCT? If so, would suggest simply stating "… and Convenience" or "… and Perceived Convenience".

- Discussion: “These findings provide strong evidence…” I would suggest tempering this statement.

- Some of the items in the Discussion section are being introduced anew and should instead be incorporated into the Results section and simply summarized as a whole in the Discussion. Specifically, patient quotations should usually appear in the Results section and new data (ie, patient statements) should not appear in the Discussion section.

- Prisma flow diagram - Seems unclear. Suggest adding a box from “Reports assessed for eligibility (n=10)” under the alternative methods heading, which states how many met inclusion criteria and were therefore added to the “studies included in review”.

VERSION 1 - AUTHOR RESPONSE

**Reviewer 1**	
Strongly recommend that the authors revise the manuscript to clearly reflect this restriction and limit their conclusions to comparisons between finger-prick and venous sampling.	Thank you for your comment. We agree that the original wording in the manuscript lacked sufficient clarity. We have amended the methods section to clarify that studies evaluating finger-prick POCT either in comparison with venous sampling or in isolation were eligible for inclusion. We have also clarified that, where comparative outcomes were assessed, venous sampling was specified as the comparator.<br><br>Also, we have acknowledged in the discussion that much of the available literature was comparative in nature and therefore the synthesis was predominantly informed by comparative perceptions of capillary versus venous testing.<br><br>(See Methods, lines 227-231/253-256, and Discussion, lines 545-549)
The authors should explain how the search strings were developed, validated, and tested. Notably, almost 30% of the included studies (6 out of 21) were not identified through the search strategy but were instead retrieved via other methods, such as manual hand searching and citation tracking. This raises concerns regarding the sensitivity of the search strategy.	Thank you for raising this. We have expanded the Methods section to clarify how the search strategy was developed and refined.<br><br>We have amended the manuscript as follows:<br>“The search strategy was developed in collaboration with a librarian using a combination of MeSH terms and keywords. Search strings were piloted and iteratively refined to maximise sensitivity.:<br><br>(See Methods, line 303-305)<br><br>We have also acknowledged this as a limitation in the Discussion:

	"A proportion of included studies were identified through citation tracking and supplementary searching, suggesting that some relevant studies may not have been fully captured through database searching alone."<br><br>(See Discussion, lines 556-558)
Figure 1: The figure misses the number of excluded studies after abstract screening.	Thank you identifying this omission. We have revised Figure 1 to include the number of studies excluded following title and abstract screening.
In the abstract, the authors state that "Medline, Embase, PsycINFO, CINAHL, Cochrane, and Web of Science were searched from inception to July 2025." However, the detailed database search strings provided in the Supplementary Material indicate that the searches were conducted in January 2024. The authors should clearly and consistently report the timing of the database searches in both the abstract and the Methods section.	Thank you for highlighting this. We have amended both the abstract and methods section to clarify that the original search was conducted from inception to January 2024 and updated in July 2025.<br><br>We have amended the manuscript as follow:<br>"We searched Medline, EMBASE, PsychINFO, CINAHL, Cochrane Library, and Web of Science from inception to January 2024. The search was updated in July 2025 to identify additional eligible studies published since the initial search"<br><br>(See Abstract, line 30 and Methods, lines 301-303)
**Reviewer 2**	
The authors do not seem to have enough knowledge on the issue of POCT and some statements (e.g the reduction of TAT and improved effectiveness) are not evidence-based and references not appropriated.<br>The question about capillary versus venous sampling is not limited to POCT and in general it requires much more and deeper concern	Thank you for this important comment. we have revised the introduction and discussion to ensure statements relating to turnaround time, effectiveness, and implementation are appropriately cautious and supported by relevant references. We have also clarified that the distinction between capillary and venous sampling extends beyond POCT implementation alone and includes broader analytical and clinical considerations.<br><br>(See Introduction, lines 106-109 and Discussion, lines 363-424)
**Reviewer 3**	

Higher User-Dependent Variability studies like 1. Operator Inexperience: Unlike laboratory technicians, POCT is often performed by untrained or multi-tasking staff, increasing the risk of errors in the pre-analytical phase (e.g., inadequate sample volume, poor technique).<br>- studied related to Procedural Inconsistency<br>- Studies of blood donation screening where point-of-care testing is primarily used at the initial stage.<br>- Studies which are showing limitations of point of care compare quality standards testing.	Thank you for highlighting these important operational considerations. We have expanded the discussion section to provide a more balances interpretation of implementation-related challenges associated with POCT, including operator variability, workflow inconsistency, training requirements, calibration, and quality assurance procedures.<br><br>These additions were incorporated as contextual consideration relevant to implementation and interpretation of acceptability findings. importance of considering operational and quality-related limitations of POCT.<br><br>(See Discussion, lines 363-424)
**Reviewer 4**	
This is one of the best reviews, on any topic, that I have critiqued! I have no comments or suggestions	We sincerely thank the reviewer for their generous and encouraging comments.
**Reviewer 5**	
The authors mention that they synthesized quantitative data as well, but this is not apparent in the Results section. The absence of quantitative data pooling is notable. Some individual study quantitative data are described, but the data were not pooled.<br>- What was the reason the authors focused solely on studies that reported qualitative findings?<br>- What about studies that reported proportion of patients and clinicians who found POCT acceptable and to what degree?<br>- Were these studies excluded unless they reported qualitative data? If so, what was the rationale for this?<br>- There could have been an opportunity to pool quantitative data regarding acceptability across studies and perhaps even compare across care settings.	Thank you for your comment. we have expanded the methods and results section to clarify extraction and synthesis of quantitative findings quantitative, qualitative, and mixed-methods studies were eligible for inclusion.<br><br>We have added a dedicated quantitative findings section in the results and a structured supplementary table summarising quantitative outcomes and direction of preference across studies.<br><br>Due to the substantial heterogeneity in study designs, populations, outcome measures, and reporting formats, formal meta-analysis was not considered appropriate. Quantitative findings were therefore synthesised descriptively and integrated withing the thematic framework through narrative synthesis.<br><br>(See Methods, lines 516-525 and Results, lines 600-621)
What was the rationale for limited the search to English language articles? With the technology now available to support translations, there is a push towards SRMAs not being limited to English language articles.	Thank you for raising this point. We have clarified in the methods section that studies were limited to English-language publications due to feasibility and resource constraints and have acknowledge this as a limitation in the discussion section.<br><br>We have amended the manuscript as follows:

	"Only studies published in English were included due to feasibility and resource constraints. This restriction may have excluded relevant non-English studies and is acknowledged as a limitation."<br><br>(See Methods, lines 246-248 and Discussion, lines 442-444)
Introduction: "*Nonetheless, concerns remain regarding analyser accuracy(30),*<br>*regulatory compliance(31), and the need for regular calibration and quality control(32). Prior*<br>*reviews have often been limited to specific professional groups(32), settings(33), or*<br>*conditions(15, 34)".* I believe this is the rationale statement to justify this study, but does not seem to match the intent of the systematic review. This review was specifically focused on acceptability among patients and clinicians. I would suggest keeping the preceding statement as the points still stand, and adding a subsequent statement to describe the importance of patient and clinician acceptability (in addition to those other factors listed).	Thank you for this helpful observation. We have revised the introduction to strengthen the rationale for examining acceptability specifically among patients and healthcare professionals. We now explicitly define acceptability as a multidimensional construct and clarify its importance for successful implementation of POCT in routine clinical practice.<br><br>(See Introduction, lines 111-210)
Please mention that inpatient and outpatient settings were included in the Methods section. And please specify the number of studies that focused on inpatient use of POCT vs. outpatient. This will have important implications for readers.	Thank you for your comment. We have clarified in the methods that studies from inpatient and outpatient settings were eligible for inclusion. We have also expanded the results section to specify the number of outpatients, mixed inpatient/outpatient, and clinician only studies in the review.<br><br>We have amended the manuscripts as follows:<br>"Of the included studies, fourteen were conducted in outpatient settings, four included both inpatient and outpatient populations, and three clinician-only studies were conducted in general practice or community mental health settings."<br><br>(See Methods, lines 246 and Results, lines 571-574)
Implications and Conclusions: "*Finger-prick POCT is not only acceptable but consistently preferred over venous sampling."* The authors have acknowledged that the acceptability of POCT is not consistent. Please revise this statement to reflect the findings (ie, most find it acceptable/ prefer it, but not all).	Thank you for your comment. We have revised the discussion and conclusion to avoid overstating consistency across studies to ensure findings are presented more cautiously and accurately. The revised wording now reflects that finger-prick was generally acceptable and often preferred over venous sampling in comparative

	studies, while acknowledging variability in perceptions and implementation challenges across settings.<br><br>(See Discussion, lines 2-424)
Results: “Psychological affect and convenience of sampling methods”. I believe the authors are referring to psychological effects (ie, impacts)?	Thank you for raising this. We have amended the terminology throughout the manuscript to: “Psychological impacts and perceived convenience of finger-prick testing”<br><br>(See Results, lines 19 and 208) all tables have been amended.
Same subheading as above: The term “Convenience of sampling method” is a bit unclear. Is this referring to the perceived convenience of POCT? If so, would suggest simply stating “… and Convenience” or “… and Perceived Convenience”.	Thank you for raising this, we have amended the terminology throughout the manuscript to improve clarity and consistency.<br><br>(See Results, lines 19 and 208) all tables have been amended.
Discussion: “These findings provide strong evidence…” I would suggest tempering this statement.	Thank you for your comment. We have revised the discussion to use more cautious language throughout and avoid overstating the strength of evidence<br><br>(See Discussion, lines 2-424)
Some of the items in the Discussion section are being introduced anew and should instead be incorporated into the Results section and simply summarized as a whole in the Discussion. Specifically, patient quotations should usually appear in the Results section and new data (ie, patient statements) should not appear in the Discussion section.	Thank you for this helpful comment. We have revised the discussion section to ensure descriptive findings and participant quotations are presented within the results, while the discussion now focuses on interpretation and contextualisation of findings.
Prisma flow diagram - Seems unclear. Suggest adding a box from “Reports assessed for eligibility (n=10)” under the alternative methods heading, which states how many met inclusion criteria and were therefore added to the “studies included in review”.	Thank you for your suggestion. We have revised the PRISMA flow diagram to clarify the contribution of studies identified through supplementary search methods and citation tracking.

VERSION 2 - REVIEW

**Reviewer**	**1**
**Name**	**Markwart, Robby**

**Affiliation**	**Jena University Hospital, Institute of General Practice and Family Medicine**
**Date**	**22-May-2026**
**COI**	

The authors revised the manuscript to my satisfaction.

**Reviewer**	**5**
**Name**	**Honarmand, Kimia**
**Affiliation**	**London Health Sciences Centre, Medicine**
**Date**	**25-Jun-2026**
**COI**	

Thank you for the opportunity to review an updated version of these manuscript. I appreciate that the authors have addressed my prior comments and suggestions. They have also incorporated quantitative findings, which strengthens the study. The new ‘Summary characteristics of included studies’ table is very informative and a great improvement from the prior version. The Discussion section is mostly re-written and much more compelling.

- Introduction - “The distinction between capillary and venous sampling, however, extends beyond POCT alone and includes physiological, analytical, and pre-analytical considerations that may influence test performance, interpretation, and user experience.” I’m not sure what the authors mean that the distinction extends beyond POCT alone. POCT is the topic of interest and associated with the subsequent physiological, analytical, and pre-analytical considerations. Also unclear what these implications (physiological, analytical, pre-analytical) mean.

- Results: Quantitative Findings - Where satisfaction is described across studies as exceeding 90%, suggest providing the range across studies as was done earlier in that statement.

- Discussion - Theimplementtion para between lines 746-752 discussed barriers to implementation in the inpatient setting. The factors described, namely “variations in patient acuity, workflow demands, and availability” are all factors that one would expect would **increase** rather than decrease the acceptability, feasibility, and ultimately a. In fact, those exact factors make POCT more desirable in the inpatient setting than outpatient. Are there other factors that may explain the paucity of inpatient studies? Perhaps those are not the factors the lead to how POCT is “experienced and implemented” but rather performing such a study among inpatients is less common due to the aforementioned barriers?

- Discussion: Limitations - “Fourth, studies reporting limited acceptability data without meaningful exploration of patient and clinicians experience were excluded, which may have

resulted in omission of studies reporting narrower aspects of acceptability reducing generalisability." Were such studies in fact excluded by design? I did not have that impression from the Methods section, especially now that the authors have incorporated studies that reported satisfaction/ acceptability quantitatively. How were articles that reported "limited acceptability data" identified for exclusion?

VERSION 2 - AUTHOR RESPONSE

**Reviewer 5**	
- Introduction - "The distinction between capillary and venous sampling, however, extends beyond POCT alone and includes physiological, analytical, and pre-analytical considerations that may influence test performance, interpretation, and user experience." I'm not sure what the authors mean that the distinction extends beyond POCT alone. POCT is the topic of interest and associated with the subsequent physiological, analytical, and pre-analytical considerations. Also unclear what these implications (physiological, analytical, pre-analytical) mean.	Thank you for this comment. We have revised this sentence to clarify that differences between capillary and venous sampling relate not only to the method of collection but also to physiological sample composition and pre-analytical and analytical considerations, including sample collection and handling, that may influence test performance, interpretation, clinician confidence, and user experience.<br><br>(See paragraph 2, lines 155-159 tracked changes highlighted)
**Reviewer 5**	
- Results: Quantitative Findings - Where satisfaction is described across studies as exceeding 90%, suggest providing the range across studies as was done earlier in that statement.	Thank you for highlighting this. We have revised the Results section to report the range of patient satisfaction across studies, ensuring consistency with the reporting of other quantitative outcomes.<br><br>(See quantitative findings section, line 960-961 tracked changes highlighted)
**Reviewer 5**	
- Discussion – The implementation para between lines 746-752 discussed barriers to implementation in the inpatient setting. The factors described, namely "variations in patient acuity, workflow demands, and availability" are all factors that one would expect would **increase** rather than decrease the acceptability, feasibility, and ultimately a. In fact, those exact factors make POCT more desirable in the inpatient setting than outpatient. Are there other factors that may explain the paucity of inpatient studies? Perhaps those are not the factors the lead to how POCT is "experienced and implemented" but rather performing such a study among inpatients is less common due to the aforementioned barriers?	Thank you for this helpful comment. We agree the previous wording was speculative and instead have highlighted the limited number of inpatient acceptability studies. The Discussion has been revised.<br><br>(See paragraph 9, lines 267-273 tracked changes highlighted)

- Discussion: Limitations - “Fourth, studies reporting limited acceptability data without meaningful exploration of patient and clinicians experience were excluded, which may have resulted in omission of studies reporting narrower aspects of acceptability reducing generalisability.” Were such studies in fact excluded by design? I did not have that impression from the Methods section, especially now that the authors have incorporated studies that reported satisfaction/ acceptability quantitatively. How were articles that reported “limited acceptability data” identified for exclusion?	Thank you for this comment. We agree the previous wording did not clearly explain how this criterion was applied. Our intention was not to exclude studies reporting a single acceptability outcome per se, but rather studies in which acceptability was incidental to the primary study objective (e.g., studies primarily evaluating diagnostic performance/accuracy or device reliability that included only a single pain or satisfaction question without further exploration of user experience). We have revised both the Methods and Discussion to clarify this eligibility criterion.<br><br>(See Methods ‘Exclusion criteria’ lines 436-438 highlighted and see Discussion Limitations, lines 418-422 tracked changes highlighted)
